# Sigma-1 Expression in Chronic Mouse Models of Temporal Lobe Epilepsy

**DOI:** 10.1007/s12035-026-06155-6

**Published:** 2026-08-25

**Authors:** Victoria Magna Stocker, Christian Lad, Aleksandr Markov, Heidrun Potschka, Eva-Lotta von Rüden

**Affiliations:** 1 Institute of Pharmacology, Toxicology, and Pharmacy, Ludwig-Maximilians-Universität München (LMU), Königinstr. 16, 80539 Munich, Germany; 2 Graduate School of Systemic Neurosciences, Munich Center for Neurosciences - Brain & Mind (MCN), Ludwig-Maximilians-Universität (LMU) München, Munich, Germany

**Keywords:** Antiseizure medication, Temporal lobe epilepsy, Intrahippocampal kainate model, Amygdala kindling model, Immunohistochemistry, Target validation

## Abstract

Sigma-1 is an atypical receptor protein that modulates neuronal excitability and cellular stress responses, making it a promising target for therapeutic epilepsy management. However, its expression profile following epileptogenic insults, during epileptogenesis, and following chronic epilepsy manifestation remains unclear. We investigated brain Sigma-1 expression immunohistochemically and via quantitative PCR in two chronic mouse models of temporal lobe epilepsy: the amygdala kindling and intrahippocampal kainate model. Thereby, different disease phases were considered from the early phase following an epileptogenic insult, epileptogenesis, to the chronic epileptic state with recurrent seizures. Sigma-1 expression was largely stable across the examined brain areas. Transient increases in expression intensity were observed 2 days after status epilepticus in the kainate model in the CA3 region of the hippocampus and the entorhinal cortex, whereas no persistent protein-level reductions were detected in the chronic phase. Descriptive coexpression analyses indicated Sigma-1 expression in excitatory and inhibitory neurons, astrocytes, and microglia. These findings indicate that Sigma-1 expression is preserved during epileptogenesis and chronic epilepsy, supporting the continued consideration of Sigma-1 as a potential therapeutic target in epilepsy management. The early alterations observed following an epileptogenic insult further support the rationale for investigating Sigma-1-directed interventions during epileptogenesis, including preventive antiepileptogenic approaches.

**Supplementary Information:**

The online version contains supplementary material available at 10.1007/s12035-026-06155-6.

## Introduction

Epilepsy is a complex neurological disorder characterized by recurrent spontaneous seizures, affecting approximately 1% of the general population worldwide [1]. Despite the availability of numerous antiseizure medications (ASMs) aimed at preventing seizure development (ictogenesis), resistance with failure to achieve seizure control in a relevant subgroup of patients remains an issue in the therapeutic management of epilepsy [2]. In this context, the heterogeneity of epilepsy with diverse etiologies, seizure types, and individual patient responses [3] underscores the urgent need for novel therapeutic targeting approaches that can provide more effective and personalized treatment options. Along these lines, the development of disease-directed, disease-modifying, and antiepileptogenic strategies is a key focus of current and future efforts to advance innovative approaches for epilepsy management [2].

In this context, a thorough analysis of the impact of disease states on target expression is essential for the development of new ASMs, as it provides information about potential variance in therapeutic responses and possible causes of therapeutic failure.

Target validation involves confirming that a particular molecular target is implicated in the disease mechanism, is expressed under pathophysiological conditions, and can be modulated to achieve a therapeutic effect [4]. This is particularly important because disease progression can cause structural or functional alterations in the target, such as changes in ion channel or receptor subunit expression, which may diminish the efficacy of ASMs [5, 6]. Without thorough validation, promising targets may fail to translate into effective therapies, leading to wasted resources and unfulfilled hopes for patients. Concerning expression analysis, verification of the specificity of the antibody–antigen interaction and the target-specific probes is an essential step [7, 8].

Sigma-1 has emerged as a potential target in epilepsy research due to its involvement in the regulation of neuronal excitability, neurotransmission, calcium homeostasis, neuroinflammatory processes, and cellular stress responses [9–13]. It is an endoplasmic reticulum-resident chaperone protein predominantly localized at the mitochondria-associated endoplasmic reticulum (ER) interface, where it regulates calcium signaling between the ER and mitochondria and contributes to cellular homeostasis under physiological and pathological conditions [11, 14]. Beyond its role at the ER-mitochondria interface, Sigma-1 has been implicated in modulating ion channel activity, neuroinflammatory signaling, and cellular responses associated with neuronal injury and neurodegeneration [10, 15]. Through these diverse functions, Sigma-1 is considered a key regulator of cellular resilience and neuroprotection in the central nervous system [9, 10].

Experimental evidence suggests that Sigma-1 modulation can influence seizure susceptibility and seizure severity and may exert neuroprotective and antiepileptogenic effects in preclinical models of epilepsy [16–19]. In this context, it is important to note that antiseizure effects were not consistently confirmed across different chronic models. While the therapeutic potential of Sigma-1-targeted interventions remains under investigation, accumulating evidence supports a role for Sigma-1 in mechanisms relevant to ictogenesis and epileptogenesis [12]. However, epileptogenesis and epilepsy are associated with complex cellular and network alterations [20]. This may lead to altered expression of genes and proteins that serve as target candidates [21]. These alterations can affect the efficacy of drugs, which bind to and alter the function of these target sites. Consequently, knowledge about the preservation and possible regulation of a target candidate is of utmost relevance for the development of novel therapeutic approaches.

To our knowledge, the expression profile of Sigma-1 in animal epilepsy models has not yet been characterized. Here, we aimed to investigate the cellular expression level of Sigma-1 in the intrahippocampal kainate and amygdala kindling mouse models of temporal lobe epilepsy. The amygdala kindling and intrahippocampal kainate models were selected as complementary experimental paradigms representing distinct aspects of epileptogenesis and manifest epilepsy [22]. Amygdala kindling is characterized by progressive increases in seizure severity and sustained increases in seizure susceptibility induced by repeated electrical stimulation. This provides a highly standardized, well-controlled model for studying the mechanisms underlying the development of epilepsy with limited structural pathology [23]. Since the paradigm is based on repeated electrical induction of seizures, it avoids any bias from chemoconvulsants. In contrast, animals in the intrahippocampal kainate model exhibit pronounced hippocampal pathology, neuroinflammation, network remodeling, and spontaneous epileptiform activity, thus mimicking key features of temporal lobe epilepsy [22]. As these models capture different, complementary aspects of epilepsy, using them together enables the assessment of Sigma-1 expression following an epileptogenic insult and across the disease’s distinct stages and manifestations. Using immunohistochemistry (IHC) and quantitative polymerase chain reaction (qPCR), we sought to confirm Sigma-1 expression in different brain regions involved in epilepsy and epileptogenesis and to analyze its expression at the cellular level. This approach enables precise regional and subcellular localization of the protein, as well as quantification of its expression profile in both healthy and epileptic tissue, providing critical insight into its functional relevance.

## Materials and Methods

### Experimental Design

The amygdala kindling model and the intrahippocampal kainate model were used as complementary models of temporal lobe epilepsy, representing different stages of epileptogenesis and the chronic epileptic state. Mouse strains and sexes were selected according to established model-specific protocols, published literature, and our laboratory’s extensive experience with these epilepsy models, as both genetic background and sex are known to affect seizure susceptibility, disease progression, mortality, and overall model performance [22, 24]. Each model was therefore analyzed relative to its corresponding control group, and the results were interpreted as complementary observations rather than as a direct comparison between the two experimental paradigms.

**Fig. 1:**
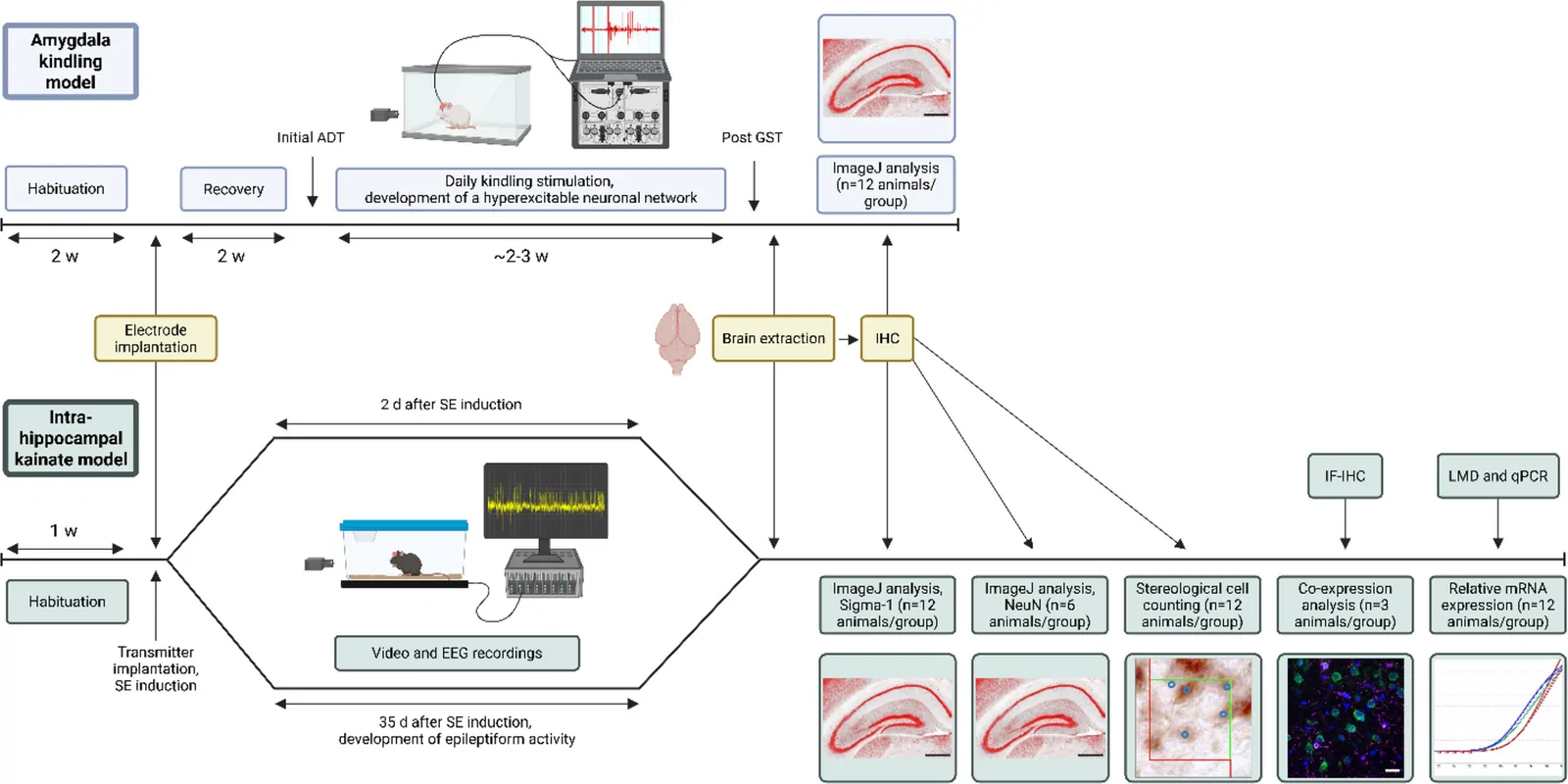
Experimental design

In the amygdala kindling model, after the arrival and habituation of the female NMRI mice (*n* = 28), a depth electrode was implanted into the right amygdala (Fig. 1). The animals underwent a 2-week convalescence phase. The initial afterdischarge threshold (initial ADT) was determined in the animals that received electrical stimulation. To induce a hyperexcitable neural network, daily suprathreshold kindling stimulations were then performed. After a total of ten generalized seizures, the animals were considered “fully kindled” and the individual afterdischarge threshold was determined again until a generalized seizure occurred (post-generalized seizure threshold; post GST). The control group was treated identically but received sham stimulations. Sixty minutes after the last induced generalized seizure, the animals were euthanized and perfused. After euthanasia, the brains were prepared for immunohistochemical analysis to determine optical density and labeled area with ImageJ in selected areas. This was intended to investigate Sigma-1 expression in an early stage of the disease. Supplementary Information (Fig. SI1) summarizes the excluded animals and the corresponding reasons for exclusion. Four animals were excluded in the course of the experiment, and the final group size for the histological analysis was 12 animals per kindling or control group. In the intrahippocampal kainate model, male C57BL/6JRj mice (*n* = 66) received a kainate injection into the CA1 region of the hippocampus after a habituation phase, inducing a non-convulsive status epilepticus (SE; Fig. 1). A depth electrode was implanted in the same region. In addition, a transmitter was inserted subcutaneously between the shoulder blades. This allowed epileptiform activity to be recorded both electroencephalographically and by video. The control group (*n* = 51 for qPCR and IHC) received a saline injection into the hippocampus and an electrode implantation. During the experiment, 21 animals were excluded, as detailed in the Supplementary Information (Fig. SI2). A total of 12 animals from either the treatment or control group were included in histological or mRNA analysis. Two days after induction of SE, the first experimental group was euthanized and perfused to investigate the early effects of SE on the regulation of Sigma-1 expression during the acute post-insult phase. The second group was euthanized and perfused 35 days after SE induction. This time point was selected to assess Sigma-1 expression in the chronic phase of the disease, after the emergence of spontaneous epileptiform events, including high-voltage sharp waves (HVSWs) and hippocampal paroxysmal discharges (HPDs). The brains of the mice intended for qPCR analysis were removed natively and immediately frozen to ensure mRNA integrity. After brain removal, specific brain areas were analyzed immunohistochemically using ImageJ (*n* = 12 per group) and stereological cell counting (*n* = 12 per group). Double and multiple immunofluorescence stainings were performed to investigate the cellular localization of Sigma-1 (*n* = 3 per group). In addition, NeuN staining was performed to detect potential effects of neurodegeneration (*n* = 6 per group). Using qPCR, the relative mRNA expression of the control group was compared with that of the kainate group at different time points (*n* = 12 per group).

### Animals

For the intrahippocampal kainate model, 9-week-old male C57BL/6JRj mice (*n* = 117; weight, 24.15 g ± 2.95 g; Janvier Labs, Le Genest-Saint-Isle, France) were single housed in type III Makrolon cages (Ehret GmbH & Co. KG, Emmendingen, Germany) upon arrival. Based on previous internal studies documenting dominance fights in group-housed males and literature, prophylactic individual housing was employed to prevent injuries related to aggression [17, 25]. In the intrahippocampal kainate model, male mice exhibited more frequent and more intense epileptiform activity after SE induction compared to female mice. In particular, HPDs occurred significantly more often in males [26]. Additionally, clear strain-specific differences were observed in the occurrence and development of epileptiform events [26]. Based on these findings, male C57BL/6JRj mice were used in the present study. For the amygdala kindling model, 8-week-old female NMRI mice (*n* = 28; weight, 29.55 ± 5.75 g; Inotiv, West Lafayette, USA) were housed in groups of four per cage until surgery. In this model, female mice were used based on our laboratory’s long-standing experience and in accordance with model-specific protocols that have been optimized and routinely applied. All cages were equipped with wood chip bedding material (SAFE® Select, J. Rettenmaier & Söhne GmbH & Co KG, Rosenberg, Germany), a plastic tunnel (Zoonlab, Castrop-Rauxel, Germany), a mouse house (Zoonlab GmbH, Castrop-Rauxel, Germany), nesting material (Zoonlab GmbH, Castrop-Rauxel, Germany), and an untreated wooden stick (Labodia, Yens, Switzerland) as additional enrichment. The animals had ad libitum access to food (Ssniff R/M diet, ssniff Spezialitäten GmbH, Soest, Germany) and tap water. All animals were housed in standardized animal rooms at a constant temperature of 22 ± 2 °C and a relative humidity of 55 ± 10%, with the light phase of the 12-h light–dark cycle lasting from 06:00–18:00 (CEST) or 05:00–17:00 (CET). Once a week, the animals were transferred to a fresh cage. The experiments were conducted in accordance with the German Animal Welfare Act and EU Directive 2010/63/EU. The mice were examined daily and scored using individual animal score sheets during the experimental phases. All animal experiments were approved by the Government of Upper Bavaria (ROB-55.2–2532.Vet_02-20–99 and ROB-55.2–2532.Vet_02-22–44). The results were processed and presented according to the ARRIVE (Animal Research: Reporting of In Vivo Experiments) guideline 2.0 [27]. All experimental procedures adhered to the principles outlined in the EQIPD Quality System Guidelines (https://eqipd.org/) to ensure standardized and consistent practices. Figure 1 provides an overview of the experimental models and methodological workflow implemented following tissue preparation.

### Amygdala Kindling Model—Surgery and Electrode Implantation

After an acclimatization phase of at least twelve days, female NMRI mice were prepared for depth electrode implantation. To prevent mutual manipulation of the surgical wound, the female mice were also kept individually postoperatively. A multimodal analgesia concept was implemented for perioperative pain management, which included buprenorphine (0.05 mg/kg, s.c., Buprenovet® Multidose, 0.3 mg/ml; Elanco Animal Health, Indiana, USA), meloxicam (5 mg/kg s.c., Metacam® 0.5% injection solution; Boehringer Ingelheim, Ingelheim, Germany), and the local anesthetic bupivacaine with epinephrine (8 mg/kg, s.c., bupivacaine 0.5% with epinephrine 0.0005%, Jenapharm®, mibe GmbH, Brehna, Germany, diluted 1:2 with 0.9% sodium chloride solution, B. Braun SE, Melsungen, Germany). Buprenorphine and meloxicam were injected 30 min before induction of anesthesia with 4% isoflurane (Isoflurane CP, CP-Pharma Handelsgesellschaft, Burgdorf, Germany), while bupivacaine with epinephrine was administered only after induction of anesthesia, 15 min before the first incision. Under maintenance anesthesia with 2% isoflurane, four trepanation holes were drilled into the skull, and a depth electrode was implanted and fixated as described before [17]. The depth electrode was implanted using the following stereotactic coordinates relative to bregma: anterior-posterior −1.0 mm, lateral +3.2 mm, and dorsoventral −5.2 mm. Representative electrode placement is shown in a thionine-stained brain section in the Supplementary Information (Fig. SI3). Postoperatively, all animals received a preheated Ringer’s lactate solution (RiLac, 10 ml/kg, s.c., B. Braun SE, Melsungen, Germany) and analgesic treatment with meloxicam, which was administered subcutaneously once daily for a further three days after the procedure.

### Amygdala-Kindling

After a 2-week recovery period following electrode implantation, the animals were randomly assigned to either the experimental group (kindling group, *n* = 12) or the control group (*n* = 12) using simple randomization (R Version 4.1.1, R Core Team, 2021). The control group received sham stimulations, exposing the animals to the handling procedures but without actual stimulations. The kindling process was carried out as described before [17, 18]. Each stimulation used a monophasic square wave pulse at 50 Hz for approximately 0.8 s. After determining the individual initial ADT, the animals were stimulated once daily with 700 µA above the threshold, 5 days a week, until each animal developed ten generalized seizures. All epileptic seizures were presented as motor seizures and verified by EEG recordings. Mice were then considered to be “fully kindled.” The following day, the seizure threshold was measured again, during which the animal exhibited a generalized motor seizure with electroencephalographic seizure activity post GST. Seizure parameters and a representative EEG pattern are included in the Supplementary Information (Fig. SI4 and SI5). Sixty minutes after determining the post GST, the kindled animals and their assigned control animals were euthanized with pentobarbital (600 mg/kg, i.p., Narkodorm® 182.3 mg/ml, CP-Pharma GmbH, Burgdorf, Germany). At least 30 min before euthanasia, analgesic pretreatment with metamizole (100 mg/kg, diluted 1:10 in 0.2% saccharin solution, Vetalgin®, MSD Animal Health GmbH, Schwabenheim, Germany) was administered.

### Intrahippocampal Kainate Model—Surgery and Electrode Implantation

During the 7-day acclimatization period, the animals were randomly assigned to either the experimental group (kainate group, *n* = 24) or the control group (*n* = 24) using simple randomization (R Version 4.1.1, R Core Team, 2021). Before and after the surgical procedure, the animals received treatment according to the analgesia protocol described for the amygdala kindling model surgery. The animals in the kainate-treated group also received local anesthesia at the incision site in the shoulder blade area for transmitter implantation. Bupivacaine without epinephrine (8 mg/kg, s.c., bupivacaine 0.5%, Jenapharm®, Mibe GmbH, Brehna, Germany, diluted 1:2 with 0.9% sodium chloride solution) was used for this purpose. Chloral hydrate (500 mg/kg, i.p., chloral hydrate, Emprove essential Ph. Eur., Merck KGaA, from VWR International GmbH, Ismaning, Germany), dissolved in isotonic sodium chloride solution, was used for general anesthesia. This anesthetic was chosen because studies indicate that inhalation anesthetics affect the induction of SE in this model and may increase its severity [26]. Careful monitoring of anesthetic depth and continuous perioperative pain management were ensured throughout the procedure. The transmitter and electrode implantation, as well as non-convulsive SE induction, were performed as previously described by Buchecker et al. [28] and Pérez-Pérez et al. [17]. Instead of kainic acid, the control animals received a sodium chloride solution. The following stereotaxic coordinates relative to bregma were used for the implantation of the depth electrode and the placement of the injection cannula: anterior-posterior −1.5 mm, lateral +2.0 mm, and dorsoventral −2.0 mm. Representative electrode placement is shown in a thionine-stained brain section in the Supplementary Information (Fig. SI3). The animals were then administered a pre-warmed Ringer’s lactate solution. Postoperative pain management with meloxicam was provided once daily every 24 h for the animals on the animal allowance AZ-02–20−99 and twice daily every 12 h for those on AZ-02–22−44, starting on the evening of the surgery and continuing for 3 days afterward. Besides analgesic care, the animals received a high-energy standard diet (Dietgel76A, Ssniff Spezialdiäten GmbH, Soest, Germany). In cases of severe weight loss, they were also offered a 10% glucose solution and, if necessary, subcutaneous injections of Ringer’s lactate solution as a depot.

### Electroencephalographic Analysis

The animals were placed on receiver plates (RPC-1, Data Sciences International, St. Paul, USA) and monitored for 24 to 48 h postoperatively using video and electroencephalographic (EEG) monitoring to capture the SE fully. Video and EEG recordings in the chronic phase were performed from day 21 after surgery (Wednesdays, Fridays, and Mondays for 24 h) to detect electroencephalographic seizure activity. The last day of recording was day 35 after surgery. The EEG signal was recorded using Ponemah® software (Ponemah® Software 5.20, Data Sciences International, St. Paul, USA) at a recording rate of 1000 Hz. The development of SE and spontaneous epileptiform activities was confirmed using video and EEG recordings as previously described by Pérez-Pérez et al. [17]. In the chronic phase, a distinction was made between three different epileptiform EEG patterns: HVSWs, HPDs, and generalized seizures [24]. Representative images of the three different epileptiform EEG patterns are shown in Fig. SI6. On 3 weekdays (Monday, Wednesday, and Friday), the animals were continuously monitored telemetrically and by video for 24 h to record and confirm the occurrence of spontaneous epileptiform activities. Brain removal was performed at two different times. In the early post-insult phase, 12 animals from the kainate and control groups were individually euthanized exactly 2 days after induction of SE. The second group was euthanized in the chronic phase, 35 days after induction of SE, and the brain tissue was then removed and prepared for further sample processing.

### Tissue Preparation

Animals were euthanized and subjected to transcardial perfusion with 0.01 M phosphate-buffered saline (PBS; NaCl, pH 7.4). This was followed by perfusion with 4% paraformaldehyde (PFA; granulated, Roth GmbH & Co. KG, Karlsruhe, Germany) in 0.1 M phosphate buffer (pH 7.4). Brains were removed, postfixed in 4% PFA for 24 h, and subsequently cryoprotected in 30% sucrose solution. Forty-micrometer-thick coronal sections were cut using a cryostat (CM3050 S, Leica Biosystems, Nussloch, Germany) in series of ten within the coordinates according to Franklin and Paxinos [29]: +1.69 mm to −4.03 mm relative to bregma, and then stored at −20 °C in a cold protection solution (mixture of ethylene glycol, 98% glycerol, and 0.2 M phosphate buffer (pH 7.4) and distilled water) until further processing. The brain tissue used for qPCR was removed from the skull after euthanasia and then frozen in 2-methylbutane (Carl Roth GmbH + Co. KG, Karlsruhe, Deutschland) and liquid nitrogen. The brains were stored at −80 °C until further processing.

### Validation of the Specificity of Sigma-1 Antibodies Using Immunohistochemistry

Given the availability of several Sigma-1 antibodies, their specificity was evaluated using a standardized 3,3′-diaminobenzidine (DAB) staining protocol, and expression patterns in wild-type (WT) and Sigma-1 knockout (KO) mice were verified. It should be noted that the term “antibody specificity” should always be understood in the context of the respective experimental method and protocol used. Therefore, the term does not refer to a method-independent binding property of the antibody, but rather to the reliability and selectivity of antibody binding observed under the described conditions. Brain tissue from homozygous Sigma-1 KO mice served as a negative control, thereby validating antibody specificity in this staining method. All antibodies were tested simultaneously using an identical protocol. Free-floating brain sections from three WT and three Sigma-1 KO mice were initially washed in Tris-buffered saline with 0.1% Tween20 (Th. Geyer GmbH & Co. KG, Renningen, Germany, TBST). Antigen retrieval was performed by incubating for 30 min in sodium citrate (pH 6) at 80 °C. After a 90-min incubation in a blocking buffer (UltraCruz®, Santa Cruz Biotechnology, Inc., Dallas, USA), the sections were treated overnight (24 h, 4 °C) with the respective primary antibody (Table 1). Negative controls were not incubated with primary antibodies. This was followed by incubation with the corresponding biotinylated secondary antibody (Table 1) for 60 min. Endogenous peroxidase activity was suppressed with 3% H₂O₂ (30% H₂O₂, Carl Roth GmbH & Co. KG, Karlsruhe, Germany) dissolved in TBST (15 min, room temperature). After a 60-min incubation with an ABC kit (1:100, P-400, Vector Laboratories, Burlingame, USA) in TBS, the brain sections were incubated for 1 min in a DAB solution prepared from DAB buffer tablets (Merck KGaA, Darmstadt, Germany) and enriched with 40 µl of a 3% H₂O₂ solution to visualize Sigma-1 antigens. The reaction was stopped with double-distilled water. After three additional washes, the brain sections were mounted on chromium gelatin-coated slides and coverslipped with Entellan® (Rapid Mounting Medium for Microscopy, Merck KGaA, Darmstadt, Germany).

**Table 1:** Overview of primary Sigma-1 and corresponding secondary antibodies, including exposure protocol information

		Host	Supplier	Order #	Lot #	Dilution
**Primary AB**	SigmaR1, polyclonal	Rabbit	Atlas Antibodies	HPA024071	14093	1:200
	SigmaR1, polyclonal	Rabbit		HPA018002	14183	1:200
	SigmaR1, polyclonal	Rabbit	Abcam	ab53852	GR3366732-1	1:150
	SigmaR1, monoclonal	Rabbit		ab253192	GR3272871-7	1:150
	SigmaR1, polyclonal	Rabbit	GeneTex/Biozol	GTX115389	44,343	1:750
	SigmaR1, polyclonal	Rabbit	Bioss	Bs-5111R	WC3226257	1:200
	SigmaR1, monoclonal	Rabbit	Cell Signaling	61994S	1	1:250
	SigmaR1	Rabbit	WG Ammer (in-house production)	N727	-	1:50
	SigmaR1	Rabbit	WG Ammer (in-house production)	N728	-	1:1,000
	SigmaR1	Rabbit	WG Ammer (in-house production)	N6229	-	1:500
	SigmaR1, monoclonal	Rat	Merck	MABN390	3482788	1:100
	SigmaR1, monoclonal	Mouse	Santa Cruz	sc-137075	J3119	1:50
**Secondary AB**	Goat anti-Rabbit	Goat	Vector	BA-1000	ZF0430	1:800
	Goat anti-Rat	Goat	Sigma/Aldrich	SAB700566	RI31714	1:800
	Goat anti-Mouse	Goat	Vector	BA-9200	ZB0801	1:800

### Validation of the Specificity of Sigma-1 Antibodies Using Western Blot

Sample preparation differs significantly for immunohistochemical and Western blot analyses. This can result in the target protein being present in different conformations. For this reason, the specific antibody binding was also validated using Western blot. The analysis was performed on both WT and KO samples (each *n* = 3). The tissue was homogenized in radioimmunoprecipitation assay buffer, and total protein concentration was determined using the Pierce™ BCA Protein Assay Kit (Thermo Fisher Scientific, Waltham, MA, USA), with absorbance measured at 562 nm on a microplate reader (BioTek Instruments, Winooski, VT, USA). Equal amounts of protein (10 µg per lane) were separated by SDS-PAGE on a 10% polyacrylamide gel (100 V for 2 h) and transferred to a polyvinylidene fluoride membrane (Thermo Fisher Scientific) using a wet transfer system at 80 V for 80 min. Transfer efficiency was confirmed by Ponceau S staining (Serva Electrophoresis GmbH, Heidelberg, Germany). The membrane was blocked for 1 h at room temperature in ROTI®Block (1:10 in TBST; Th. Geyer, Renningen, Germany), and then incubated overnight at 4 °C with the primary antibody (Table 2). After three washes in TBST, the membrane was incubated for 1 h at room temperature with a horseradish peroxidase-conjugated secondary antibody (Table 2). Chemiluminescent detection was performed using the Pierce™ ECL Western Blotting Substrate (Thermo Fisher Scientific) and imaged with a Fusion SL system (Vilber, Collégien, France). For loading control detection, the membrane was stripped using Stripping Buffer (Thermo Fisher Scientific) for 30 min at 37 °C, re-blocked as described above, and incubated with an anti-β-tubulin antibody followed by the appropriate secondary antibody and ECL detection.

**Table 2:** Overview of primary Sigma-1 and secondary antibodies used for Western blot

		Host	Supplier	Order #	Lot #	Dilution
**Primary AB**	SigmaR1, polyclonal	Rabbit	Atlas Antibodies	HPA024071	14093	1:500
	SigmaR1, polyclonal	Rabbit		HPA018002	14183	1:1,000
	SigmaR1, polyclonal	Rabbit	Abcam	ab53852	GR3341811-3	1:125
	SigmaR1, monoclonal	Rabbit		ab253192	GR3341811-8	1:1,000
	SigmaR1, polyclonal	Rabbit	GeneTex/Biozol	GTX115389	44343	1:500
	SigmaR1, polyclonal	Rabbit	Bioss	Bs-5111R	YA3800263B	1:500
	SigmaR1, monoclonal	Rabbit	Cell Signaling	61994S	1	1:1,000
	SigmaR1	Rabbit	WG Ammer (in-house production)	N727	-	1:2,000
	SigmaR1, monoclonal	Rat	Merck	MABN390	3482788	1:100
	SigmaR1, monoclonal	Mouse	SantaCruz	sc-137075	J3119	1:50
	ß-tubulin	Mouse	Thermo Fisher	MA5-16308	XG349243	1:5,000
**Secondary AB**	Goat anti-Rabbit		Abcam	ab97080	GR3342713-2	1:10,000/ 1:20,000
	Goat anti-Rat		Jackson/Biozol	112–035–167	156218	1:10,000
	Goat anti-Mouse		Jackson/Biozol	112–035–062	156325	1:10,000

### Immunohistochemical Staining of Sigma-1

None of the antibodies resulted in specific Sigma-1 detection using the previously described protocol. Literature research led to a protocol by Liu et al. [30], which showed a specific expression pattern in immunofluorescence detection when using a 1% SDS solution (Carl Roth GmbH & Co. KG, Karlsruhe, Germany) in TBS for antigen retrieval [30]. Therefore, the original protocol was adjusted for immunohistochemical detection using DAB. However, upon re-evaluation, a different antibody (HPA018002, Atlas Antibodies, Stockholm, Sweden) was used compared to the original protocol. With these adjustments, specific antibody binding to the Sigma-1 antigen was successfully detected.

Free-floating sections were washed multiple times in TBST between procedural steps. Antigen retrieval was performed for 10 min using a 1% SDS solution in TBS. Afterwards, the sections were blocked in a solution of 0.5% Triton X-100 and 1% fish gelatin in TBS. The Sigma-1-specific primary antibody (1:200, HPA018002, Atlas Antibodies, Stockholm, Sweden) was incubated overnight (24 h at 4 °C). The next day, sections were incubated for 90 min with the biotinylated secondary antibody (1:800, BA-1000, Vector Laboratories, Newark, USA). Endogenous peroxidase was blocked with 3% H_2_O_2_ in TBS for 15 min at room temperature. After a 60-min incubation with an ABC kit in TBS (1:100, P-400, Vector Laboratories, Newark, USA), the sections were incubated with 3,3′‑diaminobenzidine (DAB) solution for 1 min before the reaction was stopped by rinsing with double-distilled water. A negative control underwent the same steps, except that it was incubated without the primary antibody. The brain sections were then mounted on slides with chromium gelatin and covered with Entellan® (Rapid Mounting Medium for Microscopy, Merck KGaA, Darmstadt, Germany). To confirm the localization of the electrodes in the amygdala and CA1 region of the hippocampus, staining with thionine acetate (Sigma-Aldrich, St. Louis, USA) was performed. Representative electrode placement for both models is shown in thionine-stained brain sections in the Supplementary Information (Fig. SI3).

### Determination of Optical Density and Labeled Area of Sigma-1-Positive Cells

The open-source software ImageJ (imagej.nih.gov/ij/) was used to measure the optical density and the labeled area of Sigma-1 expression based on thresholding in the amygdala-kindled and kainate-treated animals, as well as their respective control groups. Optical density was used as a measure of staining intensity within the Sigma-1-immunoreactive structures, whereas the labeled area reflected the spatial extent of detectable Sigma-1 immunoreactivity. These parameters provide complementary information on the immunohistochemical staining pattern but do not represent direct quantitative measures of total Sigma-1 protein abundance.

To determine suitable euthanasia time points, a preliminary study analyzed optical density and labeled area across different regions (parietal cortex, piriform cortex, CA3 region, and hippocampal hilus). For this purpose, two kainate-treated animals per time point (24 h and 48 h after SE induction) were compared with a control animal, which was also euthanized 48 h after electrode implantation. In the kindling model, the timing was determined based on three animals examined at 1, 6, and 24 h after post GST. The highest values for optical density in the examined brain regions were recorded 48 h after SE induction and 1 h after the post GST determination. Based on these results and earlier studies in which a stress-induced increase in the expression of Sigma-1 and other chaperone proteins was observed within a few hours [31–33], these two time points were selected for euthanasia in the main study. The analysis focused on brain regions associated with structural and histological changes in epilepsy research. In the hippocampus, hippocampal sclerosis with neuronal loss in CA1, CA3, and the hilus of the dentate gyrus, accompanied by moss fiber sprouting and recurrent excitatory circuits, represents the central histopathological finding of mesial temporal lobe epilepsy (MTLE) [34, 35]. The loss of hilar mossy cells and somatostatinergic interneurons significantly weakens translaminar inhibition in the dentate gyrus and is considered an important mechanism of disinhibition and increased excitability of granule cells in temporal lobe epilepsy [36–38]. The amygdala is established as a primary seizure focus due to its low kindling threshold and the selective loss of somatostatinergic interneurons following seizure activity [39]. The entorhinal and perirhinal cortices, along with the hippocampus, form a reverberating rhinal-hippocampal loop. The abnormal amplification of this loop is a characteristic feature of temporal lobe epilepsy [40]. Preferential neuronal loss in layer III of the entorhinal cortex is a well-documented histological finding [41]. The piriform cortex acts as a generator and amplifier of limbic seizures [42]. Thalamic nuclei, including the mediodorsal, lateroposterior, and reticular nucleus, coordinate large-scale synchronization of epileptic discharges. The mediodorsal nucleus shows a preferential atrophy in MTLE, while the lateroposterior nucleus inhibits seizure spread [43, 44]. GABAergic neurons of the reticular nucleus mediate a central desynchronization mechanism, whose loss promotes thalamic hypersynchrony [45]. The involvement of the parietal cortex is supported by imaging and neuropathological findings demonstrating bilateral frontoparietal cortical thinning in MTLE, a consequence of secondary seizure spread via thalamocortical networks [46, 47].

Analysis covered the full extent of each brain region without further subdivision into individual layers or nuclei. For each region, mean values were derived from two to three regions of interest sampled at anterior, central, and posterior bregma levels. The parietal cortex was assessed only at central coordinates. Details of the number of analyzed sections within the areas and their bregma coordinates are included in Table 3. Following the same protocol, the respective regions were also microdissected and then analyzed by qPCR.

**Table 3:** List of brain areas analyzed using ImageJ to assess the optical density and labeled area of Sigma-1 expression

Area	Distance from bregma (mm)	Amygdala kindling model		Intrahippocampal kainate model	
		Ipsi-lateral	Contra-lateral	Ipsi-lateral	Contra-lateral
**Hippocampus** CA1, CA3 (*Stratum oriens*, *Stratum pyramidale, Stratum radiatum*, *Stratum lucidum, Stratum lacunosum-moleculare*) and hilus of the dentate gyrus	Anterior, − 1.31 Central, − 2.03 Posterior, − 3.27	X			X
**Amygdala**	Anterior, − 0.83 Central, − 1.31 Posterior, − 2.03		X	X	
**Thalamus** Mediodorsal, lateroposterior, and reticular nucleus	Anterior to posterior, − 1.55 to − 1.91	X		X	
**Cortical areas**					
Parietal cortex	Central, − 2.03	X			X
Perirhinal cortex	Anterior, − 1.55 Posterior, − 2.03	X		X	
Entorhinal cortex	Anterior: − 3.27 Posterior: − 3.87	X		X	
Piriform cortex	Anterior, 1.69 Central, − 1.31 Posterior, − 2.03	X		X	

Images of brain areas were captured using a fluorescence microscope (BZ-X710, Keyence Corporation, Osaka, Japan). Slide labels were concealed to ensure blinding during image collection and throughout quantitative analysis. Images were captured using a ×20 objective lens with an exposure time of 1/120 s. Before determining the optical density and the labeled area, the scale was calibrated using a grid and a scale reference. A scale of 3.1800 pixels/µm was used for all measurements. The grayscale values were then calibrated. For this purpose, a Rodbard calibration was performed, in which grayscale values were measured step by step. The measured values were also used uniformly for all analyses. Brain regions were outlined using the polygon tool and converted to an 8-bit image. Images were normalized by applying a saturation threshold of 0.3 for pixel intensity. Threshold values ranging from 1 to 106 were consistently used across all animals, independent of the model or group level.

### NeuN Analysis

To rule out the possibility that changes in Sigma-1 expression were related to disease-associated neurodegeneration, staining with the neuronal cell marker NeuN was performed. For optical density and labeled area analysis, six animals were randomly selected from each group (kainate and control) at both time points. Staining was performed using the standard DAB protocol established for antibody validation. Antigen retrieval was carried out in sodium citrate at 80 °C for 30 min, followed by blocking the sections for 1 h in blocking buffer. The primary antibody used was NeuN (1:500, MAB377, Lot# 3468221, Millipore), and the secondary antibody (1:300, BA-9200, Lot# ZG1216, Vector/Biozol) was incubated for 90 min. NeuN analysis was conducted in the subregions of the hippocampus, as well as the piriform and entorhinal cortex. Detailed information on the number of analyzed slices across different areas and bregma coordinates is provided in Table 3. The quantitative analysis was performed by an investigator blinded to group allocation.

### Stereological Cell Counting

To quantify the number of Sigma-1-immunopositive cells in various brain regions per unit volume, a stereological cell counting was conducted. Therefore, brain sections were mounted at 120-µm intervals and counted using the MicroBrightField System (MBF Bioscience, Williston, USA). Based on significant changes in optical density and labeled area measurements, cell counts were performed in kainate-treated animals and controls in the CA1 and CA3 regions and hilus of the hippocampus contralateral to the electrode (bregma coordinates from −1.07 to −4.03 mm), as well as in the entorhinal cortex ipsilateral to the electrode (bregma coordinates from −1.91 to −4.03 mm). The equipment used has already been described by Nowakowska et al. [48]. During evaluation, the experimenter was blinded to group assignment. Quantification was performed using the software “Stereo Investigator” (version 2023.2.1; MBF Bioscience, Williston, USA). Areas to be analyzed were determined at ×5 magnification with 0.12 NA, while cell counting in the hilus was carried out at ×40 with 0.65 NA objectives, and in the entorhinal cortex and hippocampal regions CA1 and CA3 with a ×100 oil immersion objective. The absolute number of Sigma-1-positive cells was estimated using the optical fractionator counting method [49]. The volume of each region was determined planimetrically from the contours outlined in the workflow. Cell density was calculated as the quotient of the absolute cell number and the planimetrically measured volume (Nv = cells/mm^3^).To assess the accuracy of the stereological cell counts, the coefficient of error was calculated for all analyzed areas, which is provided in the Supplementary Information, along with stereological sampling parameters (Table SI1).

### Detection of Cellular Expression of Sigma-1 by Immunofluorescence Staining

To determine whether Sigma-1 is expressed in glial cells, the glial fibrillary acidic protein (GFAP) was used to identify astrocytes, and ionized calcium-binding adapter molecule 1 (Iba1) was employed to label microglial cells. To differentiate inhibitory and excitatory neurons, co-expression analyses were performed using glutamate decarboxylase 67 (GAD67) as a marker for inhibitory neurons and vesicular glutamate transporter 1 (vGlut1) as a marker for excitatory neurons. Additionally, the pan-neuronal marker NeuN was used to identify neuronal cell nuclei. Staining was performed on free-floating sections from three randomly selected brains per group. These included control and kainate-treated animals at 2 and 35 days after inducing SE in the intrahippocampal kainate model. Sections were washed in PBST, permeabilized with 1% SDS (1% SDS, Carl Roth GmbH & Co. KG, Karlsruhe, Germany) for 10 min, and blocked in a solution containing 0.5% Triton X-100 and 1% fish gelatin (Biotium, Fremont, CA, USA) in PBS. Multiple washing steps in PBST were performed between the outlined steps. Primary antibodies were applied for 24 h at 4 °C. After incubation with the corresponding secondary antibody the next day, sections were exposed to one or two additional primary antibodies for another 24 h at 4 °C, depending on the host species. On the third day, secondary antibodies were applied for 1 h at room temperature. NeuN-labeled sections were mounted on chromium gelatin–coated slides and coverslipped using ROTI®Mount FluorCare (Carl Roth GmbH & Co. KG, Karlsruhe, Germany). Slides were sealed with nail polish the following day. Sections stained for glial markers were additionally incubated for 30 s in Hoechst solution (BisBenzimid H 33342, Invitrogen, Waltham, USA) prior to mounting. Detailed antibody information is described in Table 4.

**Table 4:** List of primary and secondary antibodies used for immunofluorescence staining

		Host	Supplier	Order #	LOT #	Dilution
**Primary antibody**	Sigma-1	Rabbit	Atlas	HPA018002	14183	1:200
	NeuN	Mouse	Merck/Millipore	MAB377	3468221	1:500
	Gad67	Rabbit	Invitrogen/Thermo Fisher	PA5-21397	Yl404640	1:2,000
	vGlut1	Mouse	NeuroMab	75-066	472-1JU-13	1:1,000
	GFAP	Goat	Abcam	ab53554	1071213-1	1:1,000
	Iba1	Goat	Abcam	ab5076	GR273528-2	1:500
**Secondary antibody**	Alexa Fluor 488	Goat anti-Rabbit	Invitrogen/Thermo Fisher	A-11008	2521157	1:1,000
	Alexa Fluor 405	Goat anti-Mouse	Invitrogen/Thermo Fisher	A-31553	2491371	1:500
	Alexa Fluor 568	Goat anti-Rabbit	Invitrogen/Thermo Fisher	A-11036	2447870	1:500
	Alexa Fluor 546	Goat anti-Mouse	Invitrogen/Thermo Fisher	A-21123	2438513	1:1,500
	Cy3	Donkey anti-Rabbit	Jackson/Dianova	711-165−152	131748	1:200
	Alexa Fluor 594	Donkey anti-Goat	Invitrogen/Thermo Fisher	A-11058	2641989	1:1,000

### Analysis of Cell Type–Specific Expression of Sigma-1 Using Immunofluorescence Staining

To qualitatively investigate the subcellular expression of Sigma-1, images of the stained sections were captured using a confocal laser scanning microscope (Zeiss LSM 880, Carl Zeiss Microscopy GmbH, Jena, Germany). First, the presence of Sigma-1-positive cells in brain sections on the ipsilateral and contralateral sides of the electrode was visually assessed in a blinded manner across all brain regions to gain an unbiased overview of the Sigma-1 expression pattern. Then, Sigma-1 expression was specifically analyzed in areas that were also stereologically cell-counted (hippocampal subregions and the entorhinal cortex). Single-plane overview images were taken using a ×20/0.8 NA objective of the contralateral hippocampal regions and the ipsilateral side (relative to the electrode) of the entorhinal cortex. Additionally, individual image planes were captured by creating a Z-stack using a ×63/1.4 NA plan apochromatic lens (NA 1.4), which enabled high-resolution, multidimensional visualization of Sigma-1-positive cells and qualitative assessment of cellular co-expression in representative cells. The transmitted light photomultiplier tube (T-PMT) function of the microscope was also utilized to detect transmitted light, in addition to the fluorescence signal. This allowed for the visualization of the neuronal cell membrane’s structure and provided additional insights into the co-expression of Sigma-1 with the neuronal markers used in individual neurons. The images were acquired using ZEN Black software (version 2.3, Carl Zeiss Microscopy GmbH, Jena, Germany) in the “Best signal” mode of the “Smart Setup.” Depending on the image, the resolution ranged from 256 × 256 to 2048 × 2048 pixels, with a pixel size of 0.09–0.10 µm and a pixel dwell time of 1.02–1.54 µs. To enhance the signal-to-noise ratio, two scans were performed per focus plane. The laser power was 1.5%, and the detector gain ranged between 750 and 850. All multiplex protocols were first established as single-stain protocols using only one primary antibody and subsequently combined in a second step to verify signal specificity and exclude relevant spectral bleed-through between fluorescence channels. For each immunohistochemical detection, a negative control was processed identically, omitting incubation with the primary antibodies, to assess non-specific secondary antibody binding and tissue autofluorescence.

### Quantitative Real-Time Polymerase Chain Reaction (qPCR)

Sigma-1 expression was analyzed in previously studied regions of the hippocampus (CA1, CA3, hilus of the dentate gyrus) and entorhinal cortex to understand regulation at the mRNA level. Therefore, 12 µm-thick coronal sections were prepared. Brain slices were mounted on a PEN membrane-coated slide (2 µm, Leica Biosystems, Nussloch, Germany) at different bregma coordinates (Table 3). Additional sections were mounted at the level of electrode implantation to verify the electrode’s location in the CA1 region. Sections were stored at −80 °C until staining. First, RNase-free thionine was applied to the slide for 1 min. The slide was then rinsed twice for 15 s in RNase-free water and subsequently soaked in a 75% ethanol solution (ethanol for molecular biology, Carl Roth GmbH & Co. KG, Karlsruhe, Germany). Next, the slide was incubated for 30 s in a 95% ethanol solution, followed by immersion in 100% ethanol. After drying, the slides were stored at −80 °C until laser microdissection (LMD) was performed. Using an LMD microscope (DM6000, Leica Microsystems CMS GmbH, Wetzlar, Germany), the regions of interest were dissected. Additionally, the parietal cortex was removed from each brain to assess mRNA integrity. mRNA integrity testing was performed exclusively as a quality control measure and was not part of the subsequent gene expression analysis, which was conducted on individual samples from individual animals. For quality control, the parietal cortices from four brains were pooled and analyzed. The quality of all pooled samples was within an acceptable range. Further details on RNA integrity testing and the kits used are provided in the Supplementary Information (Table SI2 and SI3). Based on literature research, several endogenous controls were selected [50–52]. In a preliminary experiment, Ct values of naive, amygdala-kindled, and intrahippocampal kainic acid–treated animals were compared to guide the selection of the two most stable endogenous controls. Across all samples, Non-POU domain-containing octamer-binding protein (NONO) and Ribosomal Protein L13a (RPL13A) (Mm01234361_g1 and Mm05910660_g1, TaqMan™ Assay, Thermo Fisher Scientific, Waltham, USA) were identified as the two most stable endogenous controls using RefFinder. The suitability of the selected reference genes was further confirmed by comparing the Ct values of NONO and RPL13A across experimental groups and time points using a two-way ANOVA, which revealed no significant differences. Before total mRNA isolation, performed with the RNAqueous™ Micro Kit (Thermo Fisher Scientific, Waltham, USA) according to the manufacturer’s instructions, all tissue samples from one brain region were pooled per brain and supplemented with lysis buffer to reach a volume of 100 µl. All parietal cortices cut on a single day (*n* = 4) were combined into one sample. mRNA was isolated from the LMD samples according to the manufacturer’s instructions. The total mRNA concentration for each sample was measured using the Qubit® 4 fluorometer and Qubit® test tubes. Standards and samples were prepared and quantified with the Qubit® RNA HS Assay Kit (Thermo Fisher Scientific, Waltham, USA) according to the manufacturer’s protocol. cDNA synthesis was performed according to the manufacturer’s protocol using the Maxima H Minus First Strand cDNA Synthesis Kit® (Thermo Scientific, Waltham, USA). Therefore, a target mRNA concentration was used for each time point and brain region (values provided in the Supplementary Information Table SI4). The reaction was incubated in a thermal cycler (SimpliAmp™ Thermal Cycler, Applied Biosystems™, Thermo Fisher Scientific, Waltham, USA) for 10 min at 25 °C, followed by 15 min at 50 °C. Finally, the reaction was terminated by heating to 85 °C for 5 min, and the concentration of the synthesized cDNA was measured using the Qubit® 1X dsDNA HS Assay Kit (Thermo Fisher Scientific, Waltham, USA) according to the manufacturer’s protocol. Additionally, a no reverse transcriptase control was prepared for the qPCR reaction. The double-stranded cDNA was stored at −80 °C until the final qPCR. In a 96-well plate (MicroAmp™ Optical 96-Well Reaction Plate, Thermo Fisher Scientific, Waltham, USA), 10 µl of TaqMan Fast Advance Mastermix (2X) (Thermo Fisher Scientific, Waltham, USA), 1 µl of the endogenous control assays for NONO and RPL13A, 0.75 µl of the Sigma-1 assay (Mm00448086_m1, TaqMan™ Assay, Thermo Fisher Scientific, Waltham, USA), and 6.25 µl of nuclease-free water were added to a 96-well plate (MicroAmp™ Optical 96-Well Reaction Plate, Thermo Fisher Scientific, Waltham, USA). Two microliters of sample cDNA was added to each reaction mixture. One triplicate was pipetted per area and endogenous control. A no-template control and the previously synthesized NoRT control were used as controls. The QuantStudio™ 3 & 5 Real-Time PCR Systems (Thermo Fisher Scientific, Waltham, USA) were used for qPCR. A total of 40 cycles were performed under the following reaction conditions: 2 min at 50 °C, 2 min at 95 °C, 1 min at 95 °C, and 20 s at 60 °C. Using 3–15 baseline cycles and a uniform standard threshold of 0.2 ΔRn, the results were displayed using QuantStudio Design & Analysis (Version 1.5.2., Thermo Fisher Scientific, Waltham, USA). Mean Ct values for the Sigma-1 assays and endogenous controls were calculated from the triplicate Ct values, and the ΔCt value was determined. If one of the three Ct values deviated by more than 0.5 Ct from the other two replicates, that value was excluded. The remaining Ct values were then averaged, provided they did not differ by more than 0.5 Ct. If this was also not the case, the qPCR was repeated on the entire sample. The difference in Ct values between the two endogenous controls within a brain area of an animal was less than 1 Ct, so the mean values of the endogenous controls and the two Sigma-1 values were calculated. To correct plate effects and ensure the comparability of results, a uniform reference cDNA served as an inter-plate calibrator. The resulting ΔΔCt values were used for statistical analysis, and the fold change was calculated as 2^−ΔΔCt^.

## Statistics

Before the experiments, a priori power analysis was performed for all experiments using the G*Power 3.1.9.2 (Heinrich Heine University Düsseldorf) [53] to determine the required sample size. A significance level of 0.05 (α error) and a power of 80% (*β* = 0.2) were used. The main target variable was the change in expression pattern with a minimum relevant biological difference of 10% in immunohistochemical analysis and 20% in qPCR. One mouse was considered one experimental unit. The collected data for each area were first tested descriptively for normal distribution using the Shapiro–Wilk test and for outliers using the Grubbs test (*α* = 0.05). In rare cases where normal distribution was not confirmed, the Mann–Whitney *U* test was consistently used as a nonparametric alternative. Outliers were excluded from both statistical analysis and graphical representation. The laboratory methodological approaches in the intrahippocampal kainate model were statistically analyzed using a two-factorial analysis of variance (ANOVA) with Šídák testing for multiple comparisons. Each kainate-treated group was compared with the control group at the respective time point. With an effect size of *f* = 0.5, the total sample size was 48 animals, with 12 animals per group. Exploratory correlation analyses between stereological cell counts and seizure parameters, as well as between ImageJ-based quantification and seizure parameters in the kainate model, were assessed using Spearman’s correlation. In the kindling model, analysis was conducted using a two-tailed Student’s *t*-test to compare means between two independent groups. An effect size of *d* = 1.22 indicated a group size of 12 animals each. All data are presented as mean ± standard deviation with significance at *p* < 0.05. Statistical calculations were performed using GraphPad Prism (10.4.1, GraphPad Software, Boston, USA).

## Results

### Selection and Validation of the Sigma-1 Antibody HPA018002 for Subsequent Expression Analysis

**Fig. 2:**
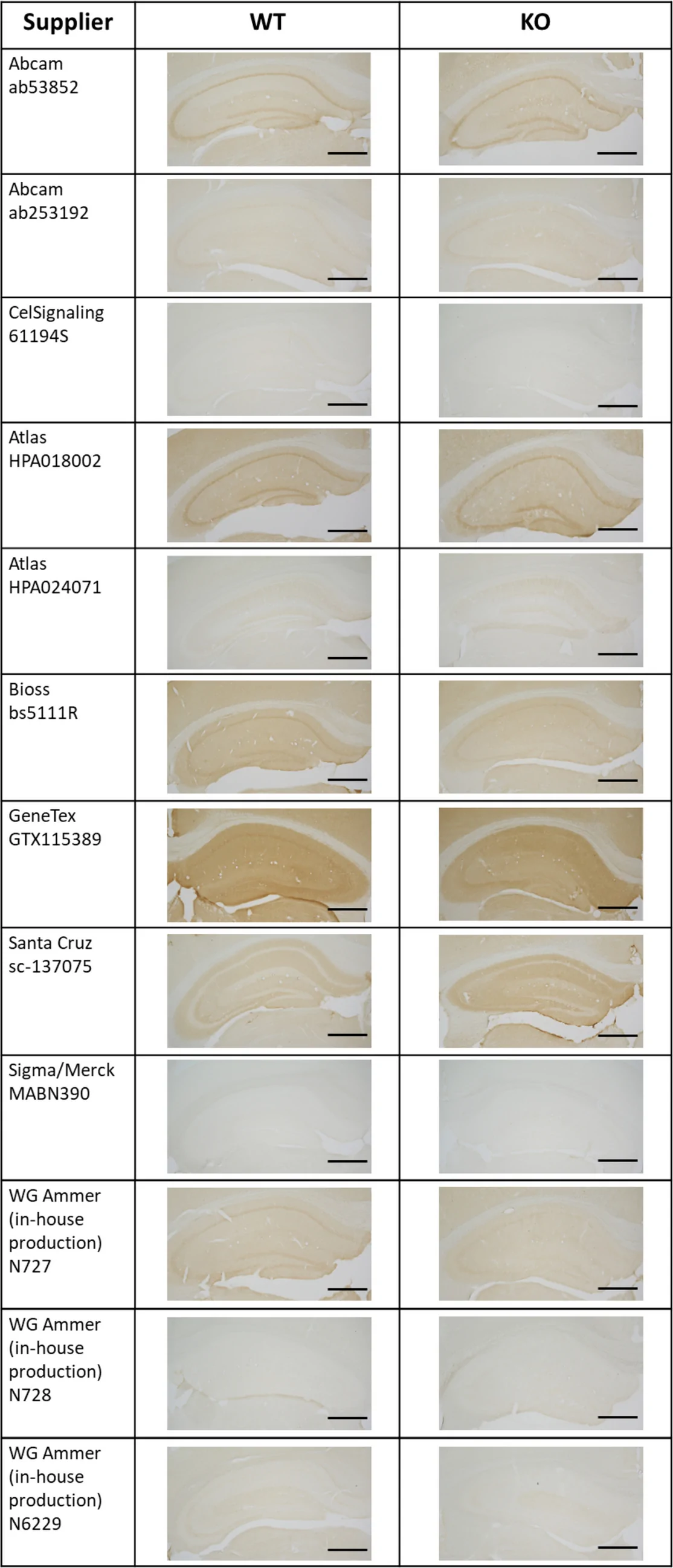
Immunohistochemical validation of commercially available and in-house Sigma-1 antibodies using hippocampal tissue from wild-type (WT) and Sigma-1 knockout (KO) mice. Representative images showing immunohistochemical staining patterns obtained with different anti-Sigma-1 antibodies, applied using a standardized staining protocol in WT and Sigma-1 KO mice (each *n* = 3). All tested antibodies produced either similar nonspecific staining patterns or only very weak signals in both genotypes, indicating a lack of specific Sigma-1 detection under these conditions. Scale bar, 50 µm

Previous studies have reported divergent findings regarding the cellular localization of Sigma-1 [30, 54–57]. These discrepancies may, in part, reflect an insufficient verification of antibody specificity. To explore this issue, several antibodies were tested (Fig. 2). Their antigen specificity was evaluated using tissue from Sigma-1 KO mice under a standardized protocol.

**Fig. 3:**
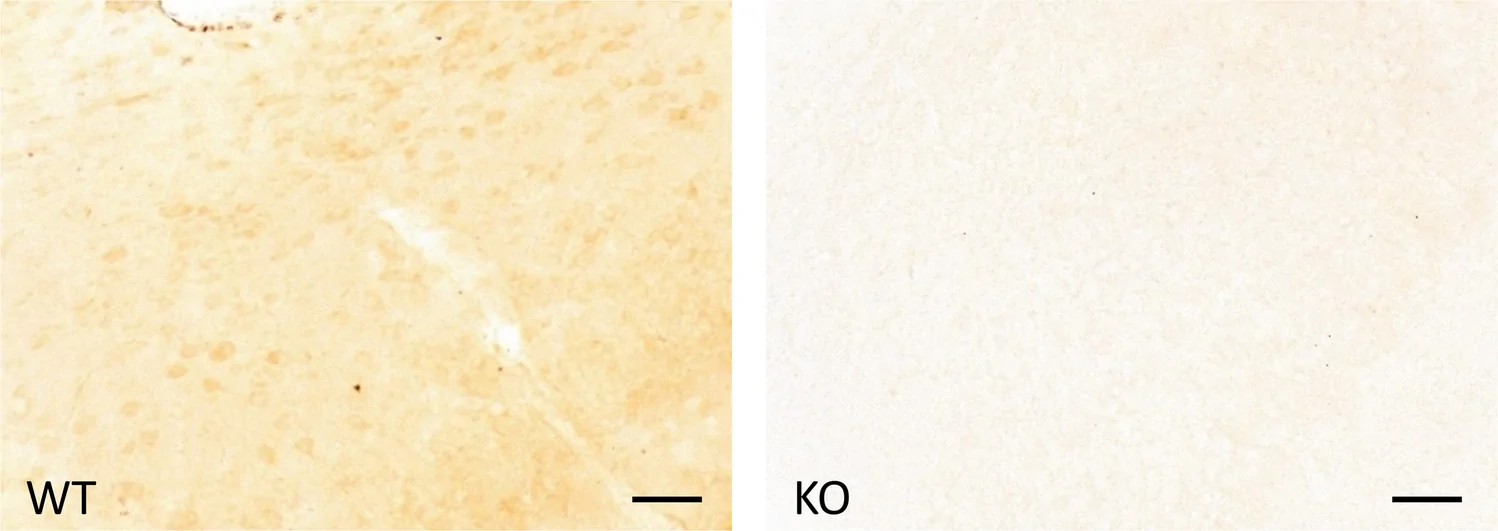
Specific immunohistochemical detection of Sigma-1 using the HPA018002 antibody. Immunohistochemical staining of the piriform cortex in a wild-type (WT) and Sigma-1 knockout (KO) mouse, demonstrating the specific detection of Sigma-1 by HPA018002 antibody. Scale bar, 20 µm

Following subsequent adjustments in line with Liu et al. [30], both immunohistochemistry using DAB and immunofluorescence staining protocols for Sigma-1 were established (Fig. 3). It is important to recognize that the term “antibody specificity” should always be interpreted within the context of the experimental method and protocol employed in each specific instance.

**Fig. 4:**
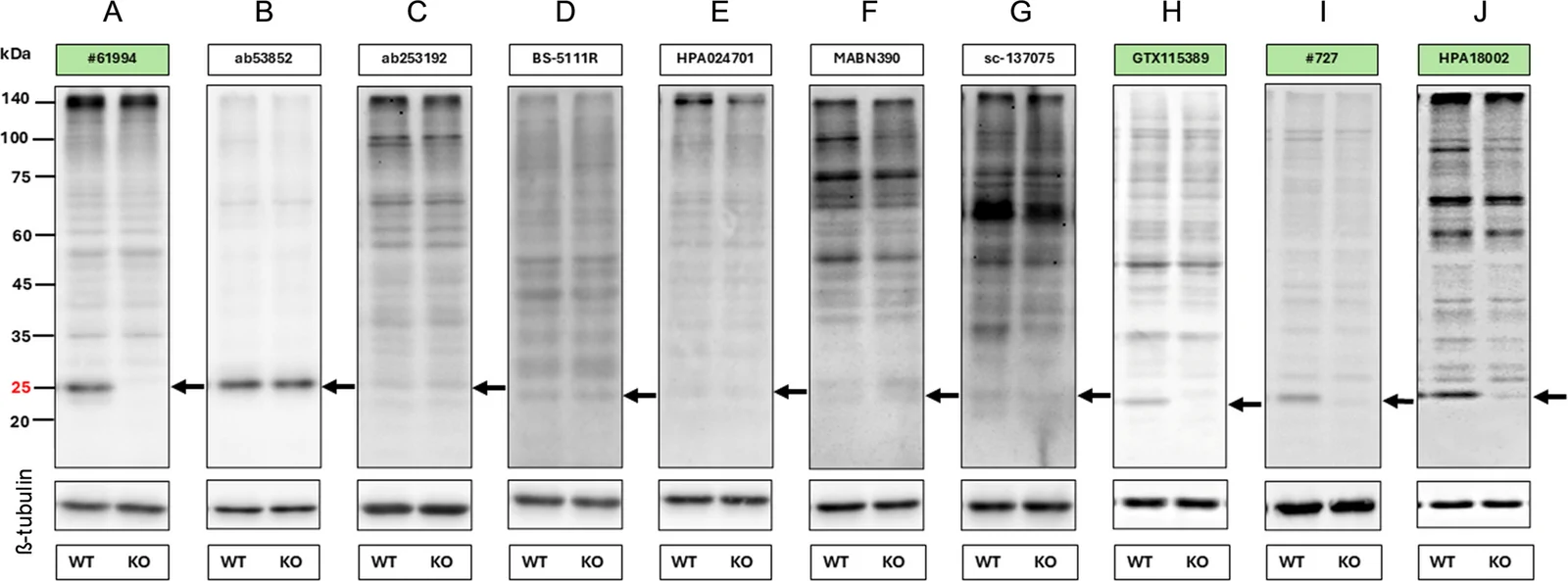
Specificity of the tested Sigma-1 antibodies in Western blotting. Each antibody was tested using three independent replicates. Representative Western blots from wild-type (WT) and Sigma-1 knockout (KO) mice are shown for each antibody. Sigma-1 (~ 25 kDa, arrow) was specifically detected by four of the ten tested antibodies (A, H–J), as demonstrated by the absence of the corresponding band in the KO samples. The antibodies presented in panel B-G yielded comparable bands at ~ 25 kDa in WT and KO samples, indicating the absence of a specific signal for Sigma-1. β-Tubulin (~ 50 kDa) served as a loading control

Based on the immunohistochemical results, ten antibodies were further evaluated by Western blot analysis. Four of these antibodies produced a specific band at the expected molecular weight for Sigma-1 (~ 25 kDa) in WT samples. In contrast, no corresponding band was observed in KO samples (Fig. 4). Notably, the antibody used for immunohistochemical analysis (HPA018002, Atlas Antibodies) also demonstrated specificity in Western blot, confirming its suitability for detecting Sigma-1.

Taken together, the immunohistochemical and Western blot analyses identified HPA018002 as the most suitable antibody for all subsequent expression analyses, as it consistently demonstrated specific detection in WT tissue and no signal in Sigma-1 KO tissue under the optimized staining conditions.

### Sigma-1 Expression Is Preserved in the Amygdala Kindling and Intrahippocampal Kainate Mouse Model

**Fig. 5:**
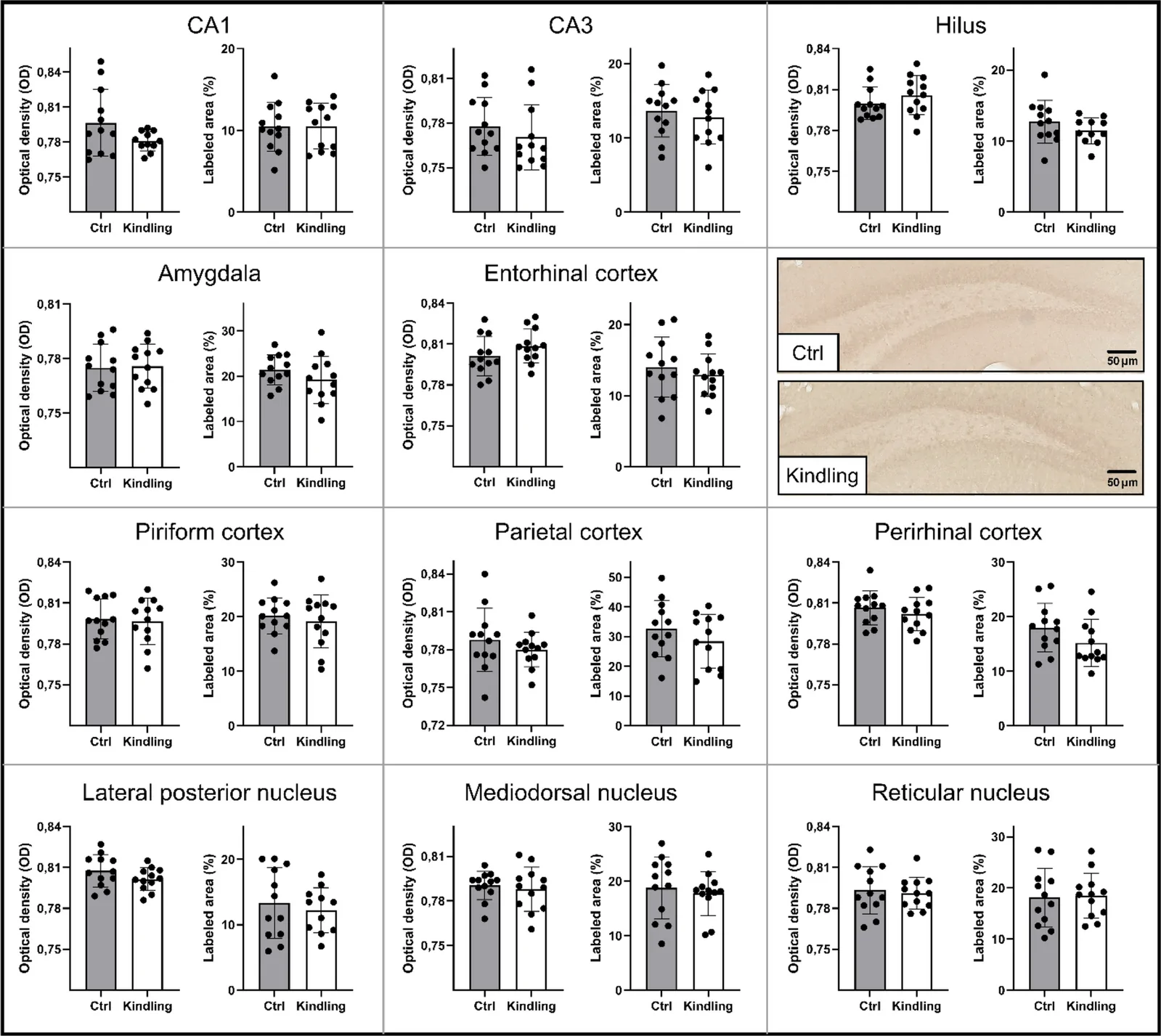
Analysis of optical density and labeled area of Sigma-1 expression in fully kindled animals and sham control animals. The results for optical density (OD) and labeled area (%) are shown for the brain regions analyzed. Sigma-1 expression in fully kindled animals was compared with that in control animals (*n* = 12 animals each). Student’s *t*-test showed no significant changes in the level of Sigma-1 expression between the two groups of animals in any of the brain areas analyzed. The two representative histological images depict the hilus of the dentate gyrus in both a control and a kindling animal. The individual data points are shown together with the mean (± SD) of the experimental groups. The Supplementary Information provides the difference between means ± SEM, 95% CI, and *p*-value (Table SI5). Except for the amygdala, all brain regions were analyzed ipsilateral to the implanted electrode. One outlier was excluded from each of the following analyses in the kindling group: optical density in the CA1 region and labeled area in the hilus and reticular nucleus of the thalamus

To determine whether and how the formation of a hyperexcitable neural network affects Sigma-1 expression, its expression was analyzed by evaluating optical density and labeled area. When comparing the kindling group with the sham control group, no significant difference was found between the two groups in the CA1 and CA3 regions and in the hilus of the dentate gyrus of the hippocampus, in the amygdala, entorhinal, piriform, perirhinal, and parietal cortices, and lateral posterior, mediodorsal, and reticular thalamic nuclei (Fig. 5).

**Fig. 6:**
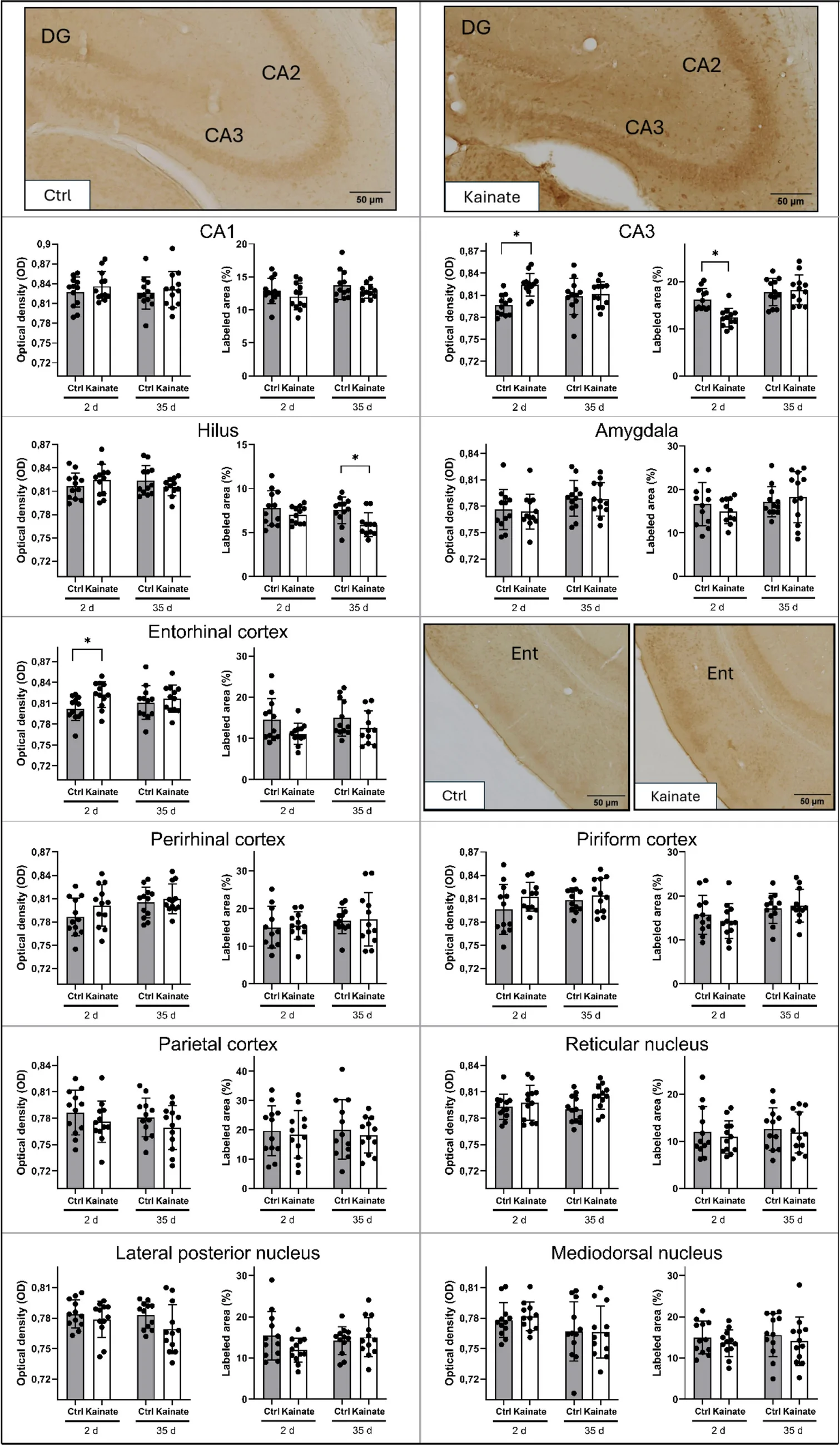
Analysis of optical density and labeled area of Sigma-1 expression in the intrahippocampal kainate model. The graphs show the optical density and labeled area of Sigma-1 expression in the analyzed brain regions. Compared to the control group, optical density in the CA3 region of the hippocampus (p_CA3_ = 0.0010) and in the entorhinal cortex (p_ent_ = 0.0359) was significantly increased in kainate-treated animals (2 days after induction of SE). At the same time, the labeled area (%) was reduced in the CA3 region (p_CA3_ = 0.0014). In the hilar region, the labeled area (%) was significantly reduced in the chronic phase (p_hilus_ = 0.0234). In all other brain areas analyzed, Sigma-1 expression levels at both time points in the experimental groups remained comparable to those in the control group. Statistical test procedures: two-way ANOVA with Šídák post hoc correction for multiple comparisons. The individual data points are shown with the group means (± SD); * = *p* < 0.05. The histological images show an increase in optical density in the kainate-treated animals compared to the control group in the CA3 region of the hippocampus and in the entorhinal cortex 2 days after induction of SE. CA1, CA2, Cornu Ammonis regions 1 and 2; DG, dentate gyrus; Ent, entorhinal cortex. The Supplementary Information provides the difference between means ± SEM, 95% CI, and *p*-value (Table SI6). Except for the hippocampal regions and the parietal cortex, all areas were analyzed on the ipsilateral side in relation to the electrode. One outlier per graph was excluded from the data analysis of the kainate group (2 d) in the labeled area of the amygdala, the optical density of the piriform cortex, and the mediodorsal nucleus of the thalamus. In the chronic kainate group (35 d), one outlier in both the optical density and the labeled area of the hilus, the labeled area of CA1, and the entorhinal cortex was excluded

SE and the associated cellular stress can significantly affect protein expression, proper protein folding, and neuronal cell death [58]. Therefore, Sigma-1 expression was also examined in the intrahippocampal kainate model at the two predefined time points following SE. In the CA1 region of the hippocampus, the amygdala, the piriform, perirhinal, and parietal cortex, as well as the lateral posterior, mediodorsal, and reticular thalamic nuclei, no significant differences were observed between the kainate and control groups in the early phase following the epileptogenic insult and following chronic epilepsy manifestation (2 and 35 days following SE). However, optical density was notably increased in both the CA3 region of the hippocampus and the entorhinal cortex during the early post-insult phase in the kainate-treated animals. In contrast, the labeled area decreased significantly in the CA3 region at the same time point. In addition, the labeled area in the hilus decreased during the chronic phase in the kainate-treated animals compared to the control group (Fig. 6).

**Fig. 7:**
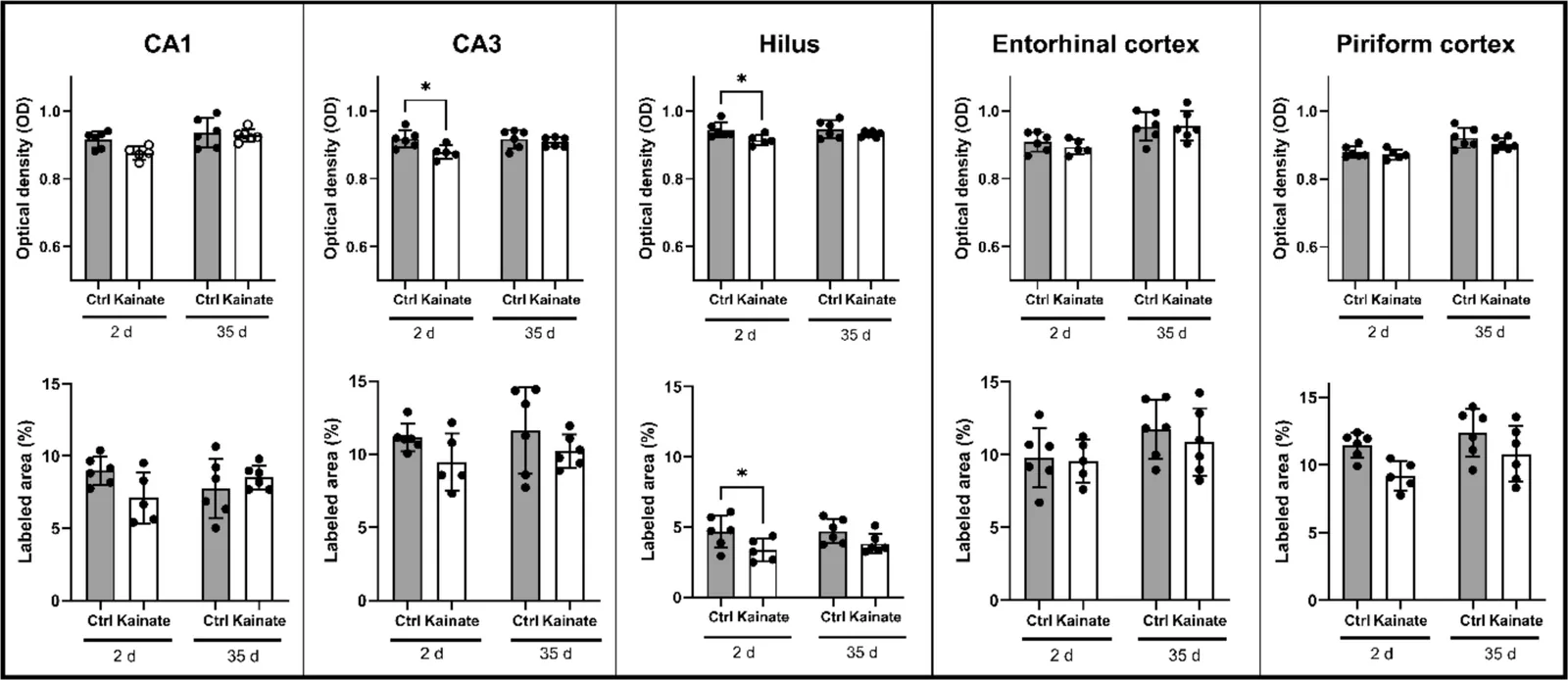
Results of the ImageJ analysis of NeuN staining. The NeuN expression showed no significant differences between the groups in the CA1 region of the hippocampus, the piriform cortex, and the entorhinal cortex at both 2 d and 35 d. However, in the CA3 region of the hippocampus, the optical density was reduced in kainate-treated animals two days after SE induction (p_CA3_ = 0.0199). In the hilus, both optical density (p_hilus_ = 0.0370) and the labeled area (p_hilus_ = 0.0498) decreased during the early post-insult phase in the kainate-treated group. The analysis indicated no significant differences between groups during the chronic phase. Since no formal sample size calculation was performed prior to these evaluations, the statistical results should be interpreted with caution. Except for the hippocampal regions, all areas were analyzed on the ipsilateral side in relation to the electrode. One animal from the control group (2 d) has been excluded from all analyses. Statistical test procedures: two-way ANOVA with Šídák post hoc correction for multiple comparisons. The individual data points are shown with the group means (± SD); * = *p* < 0.05

Kainate-induced SE leads to neuronal cell loss in the ipsilateral hippocampus in the intrahippocampal kainate model. Secondary changes have also been described in the amygdala as well as in the piriform and entorhinal cortex [44, 45]. These cell losses can subsequently be reflected in a reduction of the labeled area (%) in the analyzed brain regions. To exclude potential bias, the expression of the neuronal marker NeuN was also examined (Fig. 7).

Based on the alterations indicated by optical density and area analysis in the kainate model, Sigma-1 expression was additionally examined in subregions of the hippocampus (CA1, CA3, and hilus) and in the entorhinal cortex using unbiased stereological cell counting. For each area, the estimated cell count (Table 5) and regional volume were evaluated, revealing differences in volume between kainate and control animals, particularly during the chronic phase (Table 6).

**Table 5:** Estimated Sigma-1 cell count with standard deviation (SD)

	CA1		CA3		Hilus		Entorhinal cortex	
	Sigma-1 cells	SD	Sigma-1 cells	SD	Sigma-1 cells	SD	Sigma-1 cells	SD
**Control, 2 d**	104,054	27,392	51,840	16,530	8578	2759	41,140	18,509
**Kainate, 2 d**	130,220	40,141	48,822	13,869	11,003	2491	46,785	9282
**Control, 35 d**	109,252	42,374	48,658	22,048	6825	2828	37,686	16,214
**Kainate, 35 d**	158,950	67,583	60,409	17,698	11,671	3521	56,499	24,129

**Table 6:** Volume calculation: control vs. kainate group

Area	*p*-value (2 d)	*p*-value (35 d)
CA1	0.5517	0.0294
CA3	0.4177	0.2982
Hilus	0.1175	0.0001
Entorhinal cortex	0.8342	0.0140

**Fig. 8:**
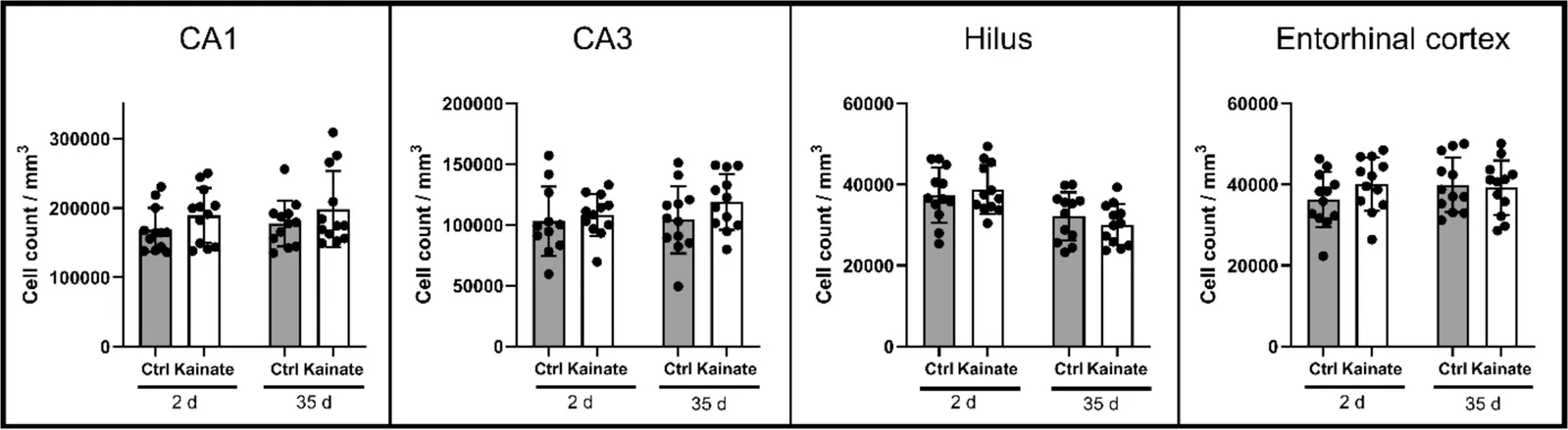
Cell density of Sigma-1 positive cells of the subregions of the hippocampus (CA1, CA3, and hilus of dentate gyrus) and in the entorhinal cortex. In all analyzed regions of the hippocampus (CA1, CA3, and hilus) and in the entorhinal cortex, a constant number of Sigma-1-positive cells per unit volume (mm^3^) was detected in both the kainate and control groups. Statistical testing: two-way ANOVA with Šídák post hoc correction for multiple comparisons. Individual data points are shown with mean (± SD) of the groups. The hippocampal regions were analyzed on the contralateral side of the electrode, whereas the entorhinal cortex was analyzed on the ipsilateral side relative to the electrode. One value in the CA3 region was excluded from the control group (35 d). The Supplementary Information provides the difference between means ± SEM, 95% CI, and *p*-value (Table SI7)

As the primary aim of this study was to assess target availability per unit volume, cell density was considered the primary outcome parameter. In these subregions, the cell density of Sigma-1-positive cells remained consistent across all groups at the studied time points (Fig. 8).

 Exploratory correlation analyses were conducted between stereological cell counts and seizure parameters, as well as between ImageJ-based quantification and seizure parameters in the intrahippocampal kainate model. No relevant correlations were found in the hippocampal regions and the entorhinal cortex. The results are presented in the Supplementary Information (Table SI8).

### Sigma-1 Expression in Neurons, Astrocytes, and Microglia

Sigma-1 expression was detected in all brain regions analyzed across both animal models and at all investigated disease stages. In a side-by-side comparison of the hemispheres, the signal appeared evenly and homogeneously distributed across all experimental groups.

**Fig. 9:**
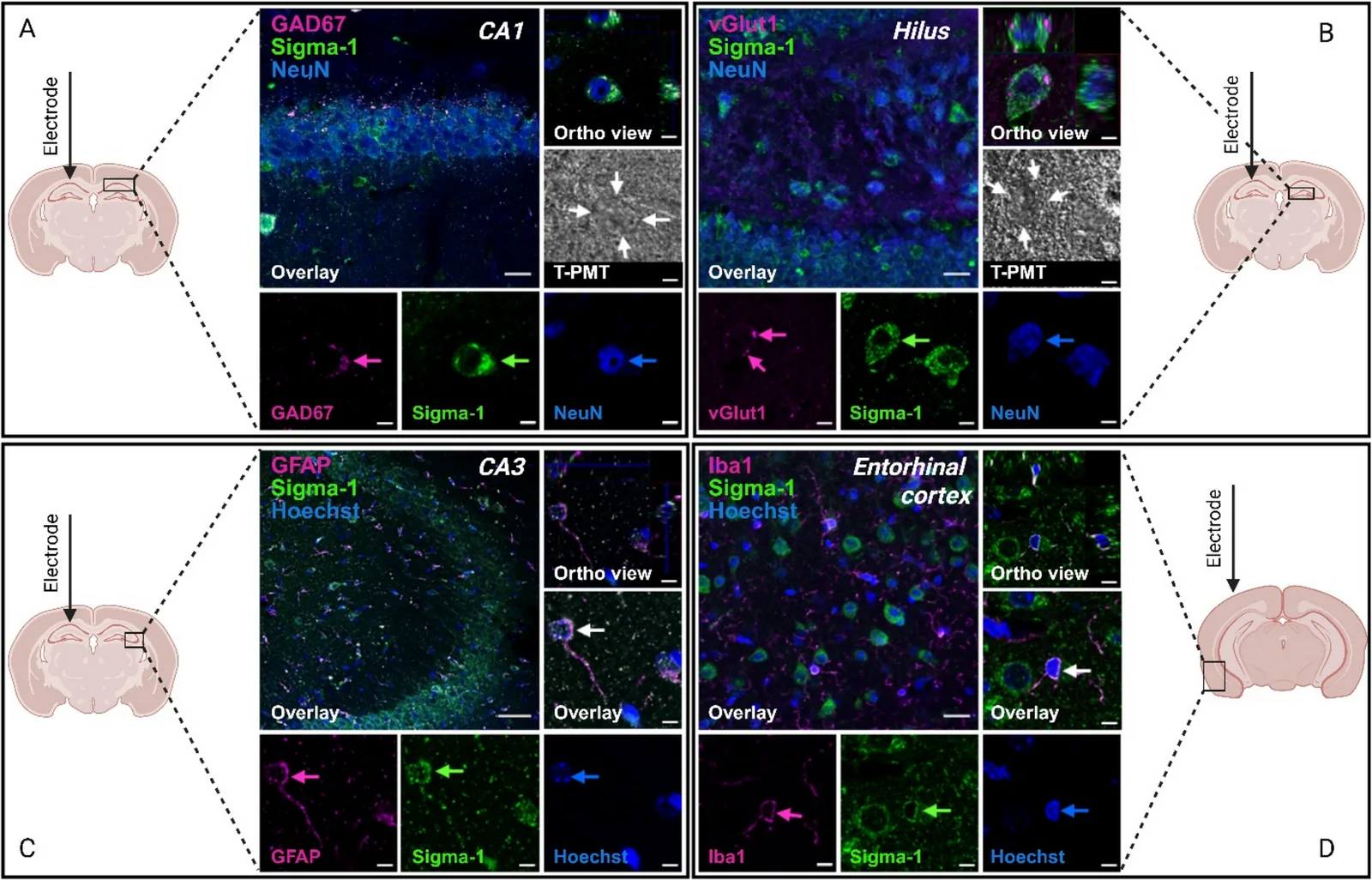
Cellular localization of Sigma-1. **A–D** Immunofluorescence multiplex staining of Sigma-1 with the cell type-specific markers GFAP, Iba1, and NeuN, combined with GAD67 and vGlut1, in the CA1 and CA3 regions, hilus of the hippocampus, and entorhinal cortex. Comparable cellular expression patterns of Sigma-1 were observed in all brain regions and experimental groups investigated. Therefore, representative double- and multiple-immunofluorescence stainings are shown. An overview image showing superimposed fluorescence signals is provided for each brain area examined (CA1, CA3, hilus of the hippocampus (contralateral to the electrode), and entorhinal cortex (ipsilateral to the electrode). Additionally, a detailed, enlarged image of representative cells is shown, with separate displays of the specific fluorescence signals of the markers used. The overview image of the CA1 region and the entorhinal cortex is from a kainate-treated animal 2 d after SE induction. The overview image of the CA3 region and the hilus of the dentate gyrus is from a control animal, 2 d and 35 d after SE induction. **A** and **B** show the cellular localization of the Sigma-1 signal (*green arrow*) alongside neuronal markers in detailed images. In the CA1 region of the hippocampus (**A**), Sigma-1 appears to be localized near the nucleus and to co-express with NeuN (*blue arrow*), a marker for neurons. Additionally, Sigma-1 shows apparent co-expression with punctate GAD67 signals (*magenta arrow*), which indicate inhibitory neurons (kainate, 35 d). In the hilus of the dentate gyrus (**B**), Sigma-1 (*green arrow*) is similarly observed in NeuN-positive cells (*blue arrow*) and appears to be co-expressed with punctate vGlut1 signals (*magenta arrow*), labeling excitatory neurons (control, 35 d). The apparent co-expression of Sigma-1 with NeuN, GAD67, or vGlut1 within a cell was additionally assessed using the T-PMT function of the microscope. **C** shows a perinuclear, cytoplasmic ring-shaped Sigma-1 signal (*green arrow*) in the CA3 region of the hippocampus, which appears to be localized within the GFAP-labeled cytoskeleton of astrocytes (*magenta arrow*) and complemented by nuclear staining (*blue arrow*) (kainate, 2 d). **D** visualizes a comparable Sigma-1 signal (g*reen arrow*) that appears to localize to Iba1-positive microglia cells (*magenta arrow*) in the entorhinal cortex, also with accompanying nuclear staining *(blue arrow)* (control, 35 d). The supplementary Z-stacks in **A–D** provide an orthogonal view of the cells and thus provide additional qualitative support for the spatial proximity of the Sigma-1 signal relative to the respective cell markers. The scale in the overview images shows 20 µm, while the detail images have a scale of 5 µm

Double and multiple immunofluorescence stainings were performed in a representative and randomly selected subset of control and kainate-treated animals at both time points to characterize the cellular localization of Sigma-1 expression. Co-labeling with NeuN and vGlut1 suggested Sigma-1 in excitatory neurons, while NeuN combined with GAD67 indicated Sigma-1 expression in GABAergic neurons. GFAP served as a marker for astrocytes, and Iba1 detection identified microglial cells. Sigma-1 co-expression was observed in both cases (Fig. 9). The cellular expression pattern of Sigma-1 was comparable across all investigated brain regions and experimental groups. Within the cell, Sigma-1 immunostaining revealed a perinuclear, ring-shaped, and punctate pattern within the cytoplasm.

### Sigma-1 mRNA Levels Remain Constant in the Hippocampus and Are Temporarily Modulated in the Entorhinal Cortex

**Fig. 10:**
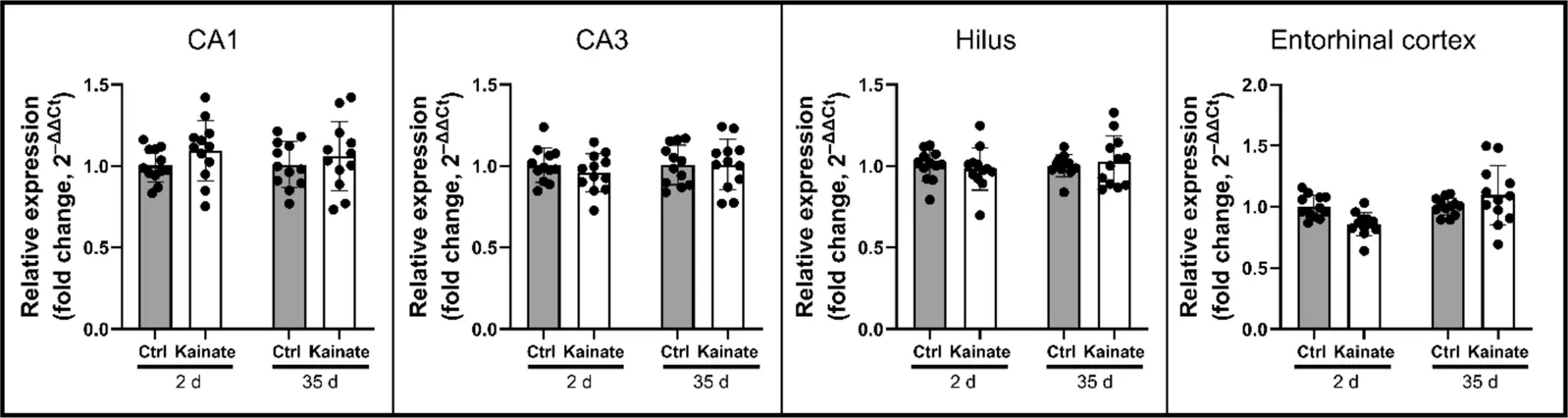
Relative expression of Sigma-1 in hippocampal subregions and entorhinal cortex. To determine the percentage reduction in expression, the fold change of the averaged ΔΔCt values was calculated. Since these are ratio values that typically do not follow a normal distribution and do not contain any information about dispersion or variance, statistical tests were not conducted. Instead, a descriptive comparison of the mean values was performed. As a result, 2 days after SE induction, the entorhinal cortex (ipsilateral to the electrode) of kainate-treated animals showed about a 14% difference in Sigma-1 expression compared to the control group, suggesting a modest, region-specific transcriptional change that was no longer detectable in the chronic phase. The subregions of the hippocampus exhibited only minor differences in mRNA expression levels contralateral to the electrode. One value of the control group (35 d) was excluded for the hilus of the dentate gyrus

To further investigate potential regional differences in the regulation of Sigma-1 expression in the kainate model, mRNA levels were determined in hippocampal subregions (CA1, CA3, and hilus) at two time points (2 days and 35 days). Relative quantification based on ΔΔCt analysis showed similar Sigma-1 mRNA levels between the kainate and control groups at both time points across all examined hippocampal regions (CA1, CA3, hilus of the dentate gyrus). In the entorhinal cortex, the assessment revealed a statistically significant decrease in Sigma-1 mRNA expression in the kainate group compared to the control group during the early post-insult phase (Supplementary Information Fig. SI7). Following the ΔΔCt method, the fold change was calculated. Two days after SE induction, Sigma-1 mRNA levels in the entorhinal cortex of kainate-treated animals were approximately 14% lower relative to the control group, representing a modest, region-specific transcriptional change that was not persistent at the chronic time point (Fig. 10).

## Discussion

This study aimed to comprehensively analyze Sigma-1 expression during epileptogenesis and disease manifestation, with a particular focus on determining whether disease-associated downregulation occurs. Establishing the stability of Sigma-1 expression is important for evaluating its suitability as a pharmacological target and understanding potential variability that could influence the effectiveness of Sigma-1-targeted therapies. Accordingly, we employed both the amygdala kindling and intrahippocampal kainate models, collecting tissue samples at defined stages of epileptogenesis and disease progression. Sigma-1 expression was analyzed using validated antibodies and probes, with both qualitative and quantitative immunohistochemical image analysis and qPCR-based mRNA quantification. This multimodal approach, combining complementary experimental models, enabled us to assess Sigma-1 expression regulation following an epileptogenic insult, during epileptogenesis, and in the chronic epileptic state. By integrating experimental models that capture distinct aspects of epilepsy, ranging from progressive seizure development to chronic epilepsy with spontaneous recurrent epileptiform events and pronounced neuropathology, we aimed to obtain a more comprehensive picture of Sigma-1 expression. When interpreting findings across these paradigms, it is important to consider that the experiments were conducted in different mouse strains and sexes. The selection of mouse strain and sexes was based on established model-specific considerations. As genetic background and sex are known to influence epileptogenesis, neuroinflammatory responses, glial activation, and other disease-related processes [26, 59], model-specific findings cannot be attributed exclusively to differences in the pathology. This should be considered when interpreting model-specific findings and represents a limitation of the present study. Additionally, this approach allowed us to explore individual differences in expression levels that may correspond to varying response profiles in the chronic phase. The present findings provide a foundation for future research into pharmacological targeting of Sigma-1 and its potential role in developing new therapeutic strategies for epilepsy.

### Preserved Sigma-1 Expression Across Different Stages of Experimental Temporal Lobe Epilepsy

Rigorous validation of the antibodies used for Sigma-1 detection formed an important methodological foundation for this study. Comparative testing of multiple commercially available and in-house antibodies revealed that reliable detection of Sigma-1 depends on both antibody selection and protocol optimization. HPA018002 was chosen for all subsequent experiments based on its specific detection in WT tissue and absence of signal in Sigma-1 KO tissue under optimized staining conditions. While validation in KO tissue strongly supports antibody specificity, factors such as tissue processing, epitope accessibility, and staining conditions can still influence antibody performance. These methodological considerations are important for interpreting the immunohistochemical findings of this study.

The immunohistochemical analyses provide complementary information on Sigma-1 immunoreactivity: optical density reflects the relative staining intensity within Sigma-1-immunoreactive structures, while the labeled area indicates the spatial extent of detectable Sigma-1 immunoreactivity. Although changes in these parameters have been associated with alterations in target protein expression or distribution [60–65], neither parameter alone directly measures total Sigma-1 protein abundance. Therefore, interpretation of Sigma-1 expression in this study is based on the combined assessment of optical density, labeled area, stereological cell counts, cell density, and qPCR analyses.

In the kindling model, no significant changes in Sigma-1 expression were detected immunohistochemically regarding optical density and the labeled area between the kindling and control groups. Our group has described histological changes and functional adaptations in the context of neural plasticity in the amygdala kindling model [66–68]. However, these alterations are less pronounced compared to other chronic epilepsy models, such as post-SE models [67, 69].

In the kainate model, Sigma-1 expression showed no differences between control and kainate groups in nearly all examined brain regions at both time points. Only three areas showed temporary changes. In the early post-insult phase, optical density increased in the hippocampal CA3 region and entorhinal cortex. At the same time, in the kainate animals, the labeled area decreased in the CA3 region and the hilus in the chronic phase. Increased Sigma-1 expression is associated with the cellular stress response, which stabilizes cellular homeostasis under pathological conditions [10, 70]. Stress-induced upregulation has been reported in various disease models, including motor neuron diseases and cerebral ischemia, where it has been associated with neuroprotective mechanisms [71–73]. These studies demonstrate that Sigma-1, as a cellular stress sensor, responds to pathological changes, including inflammation, oxidative stress, and protein misfolding. Increased Sigma-1 expression appears to be an adaptive response aimed at preserving cellular integrity and regulating degenerative processes [70, 74]. Thereby, translocation to the plasma membrane caused by cellular stress could enhance immunohistochemical detectability and suggest functional activation [75–77]. The increase in Sigma-1 optical density in the hippocampal CA3 region and the entorhinal cortex may reflect compensatory and modulatory mechanisms aiming to preserve or restore homeostasis in neuronal excitability and mitochondrial function in the early phase following the epileptogenic insult.

SE induced by intrahippocampal kainate injection can cause neuronal cell loss in the hippocampus. Secondary changes have also been observed in the amygdala, the piriform, and entorhinal cortex [78, 79]. Cell loss can be associated with a decrease in the area labeled for proteins expressed in these cells. Thus, we have additionally analyzed the expression of the neuronal marker NeuN in brain regions with a reduction in Sigma-1-labeled area. Except for the CA3 region and the hilus of the hippocampus, where a reduced optical density or labeled area was observed 2 days after SE induction in kainate-treated animals, no significant differences between the groups were detected. The observed changes in the CA3 region and the hilus of the dentate gyrus 2 days after SE induction could be explained by a stress-related modulation of NeuN immunoreactivity, as under cellular stress conditions such as excitotoxicity, NeuN can be regulated both in its expression and in its binding properties to antibodies [80, 81]. Furthermore, Lind et al. [82] demonstrated that the phosphorylation state of NeuN is subject to a complex regulatory network and significantly influences antibody recognition, which can limit detection. This interpretation is supported by the transient nature of the observed changes in this study. The normalization of optical density and labeled area during the chronic phase argues against extensive irreversible neuronal loss in the analyzed brain regions. Importantly, these findings are restricted to the contralateral hippocampal subregions and the ipsilateral entorhinal cortex. The ipsilateral hippocampus, which is known to exhibit pronounced kainate-induced neurodegeneration, was not included in the quantitative analysis, as severe lesion-associated alterations would substantially confound the interpretation of Sigma-1-positive cell counts. Therefore, the present data does not indicate the absence of neurodegeneration in the intrahippocampal kainate model, but rather a lack of evidence for irreversible neuronal loss in the regions analyzed. However, the limited sample size of the NeuN analysis warrants cautious interpretation of these findings.

The observed variations in optical density and labeled area might also reflect ligand-induced intracellular translocation of Sigma-1, which can be triggered by ER stress or calcium imbalance. Under such conditions, Sigma-1 relocates from its original position at the mitochondria-associated membrane to other subcellular compartments, including the ER, plasma membrane, nuclear membrane, and nucleoplasm [11, 83–85]. Reduced immunoreactivity of Sigma-1 in some studies has been attributed to this translocation or to conformational changes that reduce epitope accessibility, rather than to a true decrease in protein abundance [75, 86–88]. Thus, the reduction in Sigma-1-immunopositive area in selected brain regions may also reflect decreased accessibility of the Sigma-1 epitope due to translocation or conformational changes under conditions of cellular stress, rather than necessarily indicating a transient reduction in Sigma-1 expression.

The stereology-based quantitative analysis revealed no difference in cell density between the examined groups at any point, neither in the early post-insult phase nor during the manifestation of epilepsy.

Although this finding appears to contrast with labeled-area data, it should be considered that the two-dimensional quantitative assessment of the labeled area can be biased by disease-associated changes in the extracellular space. Notably, the volume calculation showed a significant increase in the hilus in kainate-treated animals with spontaneous recurrent seizures (Table 6). This finding suggests that model-associated changes in the labeled area data for the hilus primarily reflect an expansion of the extracellular space and associated volume increase of the brain region.

The qPCR analysis indicated that Sigma-1 expression is largely maintained at the transcription level. RNA integrity was assessed using pooled quality control samples rather than individual specimens (Table SI2). Although all pooled samples met predefined quality criteria and the stability of the endogenous controls supported the reliability of the measurements, individual variations in RNA integrity cannot be completely excluded.

Only the entorhinal cortex exhibited a modest reduction in Sigma-1 mRNA expression during the early post-insult phase. As this change was no longer detectable during the chronic phase of epilepsy, it appears to represent a modest and transient transcriptional alteration. It should be noted that mRNA expression does not necessarily reflect protein abundance. Post-transcriptional and post-translational modifications, along with protein turnover, play a crucial role [89]. Therefore, mRNA levels should be interpreted with caution as an indirect indicator of protein abundance. It has been reported that only approximately 40% of the variation in protein levels can be explained by the measured mRNA levels [90]. Although reduced mRNA content typically leads to lower protein biosynthesis, stress signals can specifically enhance translation and increase the stability of selected proteins [91]. In this context, it is crucial to consider the protein turnover rate, as it significantly affects the relationship between mRNA and protein levels and must be taken into account when interpreting regulatory mechanisms at the transcriptional, translational, and degradation stages [92]. To our knowledge, no published data is currently available on the protein turnover of Sigma-1. In addition to turnover dynamics, feedback mechanisms between transcriptional and protein levels may contribute to the observed downregulation [93]. One possible explanation is that, in the early post-insult phase, Sigma-1 mRNA levels may initially rise, followed by enhanced translation. Once sufficient protein levels are reached, transcriptional feedback mechanisms might contribute to a transient reduction in mRNA levels. However, this hypothesis cannot be addressed by the present data, as earlier post-insult time points were not investigated. Currently, only limited data are available on the temporal regulation of Sigma-1 at the mRNA level. Mitsuda et al. [31] showed that Sigma-1 is rapidly upregulated in vitro in response to ER stress. During a 120-min measurement of mRNA levels, the mRNA was already strongly upregulated after 30 min and reached its peak after 60 min [31]. Furthermore, studies of other chaperone proteins suggest very rapid mRNA regulation [32]. For heat shock protein 70, a stress-induced increase in mRNA expression was demonstrated within a few hours [33]. Therefore, the selection of euthanasia times represents a critical point in this study. Since the analysis performed refers to entire brain areas without differentiating between individual cell types or cortical layers, a cell-specific evaluation of the regulatory mechanisms is not possible. Furthermore, five Sigma-1 different splice variants have been described [94]. The TaqMan assay used in the present study detects all transcript variants containing the targeted exon boundaries and therefore quantifies total Sigma-1 mRNA without distinguishing between individual splice variants. Consequently, potential isoform-specific regulation cannot be assessed and may remain undetected in the present analysis.

### Sigma-1 Distribution Across Neuronal and Glial Cell Types in the Epileptic Brain

The present immunofluorescence analysis demonstrates cellular expression of Sigma-1 in inhibitory and excitatory neurons, as well as in glial cells, across all analyzed brain regions. This aligns with earlier studies reporting a widespread distribution of Sigma-1 throughout the central nervous system [30, 54, 95]. However, because the evaluation of Sigma-1 co-expression with cell type–specific markers relied on qualitative visual observation rather than quantitative image analysis, these results should be viewed as descriptive.

At the subcellular level, the characteristic ring-shaped perinuclear Sigma-1 signal in neuronal cytoplasm has already been documented in mouse models and cultured cells [11, 30]. The subcellular co-expression patterns of Sigma-1 in rodents remain contentious, as existing studies report inconsistent results regarding their localization in neurons, oligodendrocytes, and astrocytes [54, 56, 57]. While early research detected Sigma-1 expression only in neurons of the brain and spinal cord [54], Sigma-1-positive oligodendrocytes were described three years later [55]. Later, these findings were expanded, showing that reactive astrocytes can also express Sigma-1 [56]. These observations received confirmation in a study of a Parkinson’s disease model [57]. The comprehensive analysis by Liu et al. [30], which utilized an antibody validated by KO tissue, demonstrated cell type–specific expression of Sigma-1 in excitatory neurons, interneurons, astrocytes, oligodendrocytes, oligodendrocyte precursor cells, and microglia, consistent with the findings of the current study. These results underscore the importance of careful antibody validation to prevent false-positive results due to nonspecific interactions or cross-reactions with homologous proteins. Therefore, verifying antibody specificity is essential for ensuring the reliability and reproducibility of data when identifying and characterizing potential target structures [7, 8].

In excitatory neurons, Sigma-1 has been implicated in regulating neuronal excitability by modulating ion channels, neurotransmitter receptors, and ER stress signaling [13]. Experimental studies further suggest that Sigma-1 activity influences glutamatergic neuronal function and structural plasticity [96]. In inhibitory neurons, Sigma-1 has also been linked to GABAergic signaling, and Sigma-1 deficiency has been associated with reduced expression of the GABA_B_-R2 subunit and increased seizure susceptibility [12]. Beyond neurons, Sigma-1 is also expressed in glial cells, where it has been implicated in several processes relevant to epilepsy. In astrocytes, Sigma-1 activation has been shown to protect against cellular stress [97], whereas in microglia, it has been associated with anti-inflammatory effects and reduced neuroinflammatory signaling [98, 99]. Together, these findings suggest that the preserved expression of Sigma-1 across multiple cell types may allow pharmacological modulation of several cellular and molecular processes relevant to epilepsy and highlights the need for further research into the therapeutic potential of Sigma-1 targeting approaches in epilepsy.

## Overarching Conclusions and Future Perspectives

The comprehensive analysis of Sigma-1 expression at both the protein and mRNA levels revealed no relevant reduction during epileptogenesis or after the onset of epilepsy in either investigated model. As the investigated brain regions play key roles in the generation, synchronization, and propagation of epileptic activity [100], this suggests that Sigma-1 expression is maintained in key brain regions throughout epileptogenesis and chronic epilepsy, even though the present study does not directly address Sigma-1 function. Previous pharmacological studies from our group have demonstrated that Sigma-1-directed interventions can influence seizure-related outcomes in experimental epilepsy, although the underlying mechanism and the precise contribution of Sigma-1 signaling remain incompletely understood [17, 18]. The present findings complement these observations and support further validation of Sigma-1 as a therapeutic target candidate. Moreover, transient increases in Sigma-1 expression, as indicated by optical density analyses, suggest that prolonged seizure activity can trigger induction or translocation and that an early preventive and possibly antiepileptogenic approach targeting this induction phase could be particularly promising. Stable Sigma-1 expression in the chronic epileptic phase further suggests that Sigma-1 targeting could also be of interest for chronic therapeutic management of temporal lobe epilepsy.

The similar variance observed in the data between the control and kainate groups suggests there is no significant increase in heterogeneity of Sigma-1 expression following an epileptogenic insult and following epilepsy manifestation. These results in the two models of temporal lobe epilepsy argue against differential Sigma-1 expression contributing to differences in therapeutic responses to Sigma-1 modulators. This finding is of interest in the context of the target hypothesis of drug-refractory epilepsy, which has attributed therapeutic failure to alterations in expression, function, or modulation of target sites [5, 6, 101].

Future studies using advanced imaging and molecular techniques, as well as layer- and cell type–specific analyses, are crucial for a comprehensive understanding of the complex temporal regulation of Sigma-1. Methods such as live-cell imaging, fluorescent tagging, optogenetics, laser microdissection, proteomics, and metabolomics could provide further insights into the dynamic regulation and functional roles of Sigma-1 in epilepsy.

## Supplementary Information

Below is the link to the electronic supplementary material.

ESM 1 (DOCX 1.97 MB)

ESM 2 (DOCX 2.11 MB)

## Data Availability

The data are available through Figshare [10.6084/m9.figshare.33287073].
