# Supplementary material for: Sigma-1 Expression in Chronic Mouse Models of Temporal Lobe Epilepsy
Source: Mol Neurobiol. 2026 Aug 25;63(1):855. doi: 10.1007/s12035-026-06155-6 (PMC13506606; doi:10.1007/s12035-026-06155-6)
Supplement: Supplementary file 1 [file 12035_2026_6155_MOESM1_ESM.docx]

**Sigma-1 expression in chronic mouse models of temporal lobe epilepsy Molecular Neurobiology**

Victoria Magna Stocker^1^, Christian Lad^1, 2^, Aleksandr Markov^1, 2^, Heidrun Potschka^1‡^, and Eva-Lotta von Rüden^1‡^

^1^Institute of Pharmacology, Toxicology, and Pharmacy, Ludwig-Maximilians-Universität München (LMU), Munich, Germany

^2^ Graduate School of Systemic Neurosciences, Munich Center for Neurosciences - Brain & Mind (MCN), Ludwig-Maximilians-Universität (LMU) München, Munich, Germany

^‡^ These authors contributed equally as senior authors.

el.vonrueden@pharmtox.vetmed.uni-muenchen.de

potschka@pharmtox.vetmed.uni-muenchen.de

**Supplementary information**

# **Animal exclusion**

**Fig. SI1** Exclusion of animals in the amygdala kindling model

Fig. SI2 Exclusion of animals in the intrahippocampal kainate model

# **Electrode localization**

**Fig. SI3** Localization of electrodes in the amygdala kindling (A) and intrahippocampal kainate mouse model (B)

Coronal section through a mouse brain showing the electrode channel and the implantation of the electrode tip in the right amygdala (A) and the right CA1 region of the hippocampus (B). Thionin staining, scale: 100 µm.

# **EEG Analysis**

**Fig. SI4** Assessed seizure parameters in amygdala kindling and intrahippocampal kainate mouse model

In the amygdala kindling model (A), the animals received once daily 700 µA suprathreshold stimulations, five days a week, until each animal developed ten generalized seizures. During each electrical stimulation, seizure parameters were recorded, including seizure severity according to the Racine scale [1], which classifies seizure stages based on observed behavioral manifestations, as well as motor seizure duration and electroencephalographic afterdischarge duration (ADD). An example of a seizure-associated EEG in the amygdala kindling model is shown in Fig. SI5.

**Fig. SI5** Seizure-associated EEG in the amygdala kindling model: representative epileptiform afterdischarge

The baseline is interrupted by electrical stimulation. After a stimulation artifact lasting about 3–4 seconds, a characteristic seizure pattern appears, allowing differentiation between the ADD1 and ADD2 phases through an abrupt change in frequency.

In the intrahippocampal kainate model (B), video-EEG recordings were conducted during the chronic phase (days 21 to 35 after surgery) to confirm the development of spontaneous epileptiform activity. Three distinct epileptiform EEG patterns were identified: high-voltage sharp waves (HVSW), hippocampal paroxysmal discharges (HPD), and generalized seizures. Representative examples and the temporal course of these seizure patterns are shown in Fig. SI6.

**Fig. SI6** Representation of the different seizure patterns in the EEG of the intrahippocampal kainate model

| **EEG-Pattern** | **Example**  2 s |
| --- | --- |
| **HVSW** |   0.2 mV |
| **HPD** |   2 s  0.2 mV |
| **Generalized**  **seizure activity** |   0.2 mV  2 s |

**HVSWs** are characterized by at least a threefold increase in amplitude compared to the baseline, as well as a frequency of at least 2 Hz. This pattern must have a minimum duration of 5 seconds and be separated by an inter-event interval of at least three seconds.

**HPD:** The HPD pattern consists of two phases: the first phase matches the criteria for HVSWs. In the second phase, the frequency increases to at least 5 Hz while the amplitude decreases, remaining at least twice the baseline amplitude; this phase must also last at least five seconds.

**Generalized seizure activity** appears as very high amplitudes and high frequency in the EEG. The frequency exceeds 10 Hz, while the amplitude is at least three times the baseline. These seizures were additionally confirmed through video recordings.

# **Stereological cell counting**

Table SI1 Average values of the error coefficients calculated in the individual areas and stereological sampling parameters

|  | **CA1** | **CA3** | **Hilus** | **Entorhinal cortex** |
| --- | --- | --- | --- | --- |
| **CE, m = 1** | 0.04 | 0.06 | 0.07 | 0.07 |
| **Section evaluation interval** | 5 | | | |
| **Counting frame (µm)** | 26 x 26 | 28 x 28 | 43 x 43 | 36 x 36 |
| **Sampling grid (µm)** | 130 x 130 | 140 x 140 | 85 x 85 | 180 x 180 |
| **Dissector height (µm)** | 25.0 | 19.9 | 28.0 | 30.5 |
| **Guard zones: (µm)** | 2.0 | | | |
| **Area sampling fraction (asf):** | 1/16 | 1/25 | 1,849/7,225  ≈ 0.256 | 1/25 |
| **Height sampling fraction (hsf):** | 5/8 | 19.9/40 | 7/10 | 61/80 |

A CE value below 0.09 indicates a reliable and precise count [2].

# **qPCR**

**Table SI2** Results of the mRNA integrity check

| **Pool sample** | **Efficiency calculation Sigma-1 (%)** | **Efficiency calculation NONO/RPL13A (%)** |
| --- | --- | --- |
| **1** | 91.999 | 90.882 |
| **2** | 102.877 | 108.372 |
| **3** | 97.236 | 100.632 |
| **4** | 91.151 | 104.787 |
| **5** | 107.724 | 107.421 |
| **6** | 100.705 | 102.061 |
| **7** | 102.378 | 99.113 |
| **8** | 90.072 | 93.317 |
| **9** | 98.631 | 98.130 |
| **10** | 105.396 | 103.481 |
| **11** | 104.382 | 105.331 |
| **12** | 106.128 | 98.054 |

The quality of the analyzed mRNA was determined using a pooled sample consisting of four isolated parietal cortices. Subsequently, a dilution series was prepared, and amplification efficiency was calculated for both the Sigma-1 assay and the endogenous control. All pooled samples fell within the accepted efficiency range of 90-110% [3].

**Table SI3:** Overview of commercial kits and reagents used for qPCR

| **Kit/Reagent** | **Supplier** | |
| --- | --- | --- |
| Maxima H Minus First Strand cDNA Synthesis Kit with dsDNase | Thermo Fisher Scientific, Waltham, USA, Lot#3044674, #2807287 | |
| Qubit® RNA HS Assay Kit | Thermo Fisher Scientific, Waltham, USA | |
| RNAqueous^TM^-Micro Kit | Thermo Fisher Scientific, Waltham, USA, Lot#26246684, #01336178, #2840861, 2864980 | |
| RPL13A, Mm05910660_g1, LOT# P241030-002 TaqMan™Assay | Thermo Fisher Scientific, Waltham, USA | |
| Sigma-1, Mm00448086_m1, TaqMan™Assay | Thermo Fisher Scientific, Waltham, USA | |
|  | Target | Gen Sigmar1 (sigma non-opioid intracellular receptor 1) |
|  | Species | mouse |
|  | Catalog-/Lot# | Cat# 4351370 75; Lot# P250516-000 H10 |
|  | Design info | _m1-Assay (Probe over exon-exon junction, no gDNA amplification) |
|  | Amplicon length | ~72 bp |
|  | Target Region & Transcripts | recognized among other RefSeq transcripts NM_001286541.1 (Junction Exon 2–3), NM_001286551.1 (Exon 3–4), NM_011014.3 (Exon 3–4). |
| TaqMan^TM^ Fast Advanced Master Mix (2X) | Thermo Fisher Scientific, Waltham, USA, Lot#3007145, #3047298 | |

**Table SI4:** Target concentration of mRNA in different areas over time, used for cDNA synthesis

| **Area** | **Timepoint** | **Target concentration of mRNA in (ng/µl)** |
| --- | --- | --- |
| **CA1** | Acute post-insult phase (2 d) | 15.96 |
| **CA3** |  | 33.95 |
| **Hilus** |  | 8.96 |
| **Entorhinal cortex** |  | 29.4 |
| **CA1** | Chronic phase (35 d) | 28.14 |
| **CA3** |  | 41.3 |
| **Hilus** |  | 10.64 |
| **Entorhinal cortex** |  | 42.0 |

**Fig. SI7** Mean ΔΔCt values of Sigma-1 mRNA expression using NONO and RPL13A as endogenous controls

The ΔΔCt values for Sigma-1 mRNA expression are presented as the mean of both controls (NONO and RPL13A) across the examined brain regions. In the entorhinal cortex, kainate-treated animals exhibited significantly reduced Sigma-1 mRNA levels compared to controls (p_ent_ = 0.0149). Statistical analysis was performed using two-way ANOVA with Šídák's multiple-comparison test. Individual data points are shown with group means (± SD), with * indicating p < 0.05.

# **Additional quantitative estimates**

**Table SI5** Differences between means ± SEM or medians (Hodges-Lehmann), with 95% CI and p-value, for the analysis of optical density and labeled area of Sigma-1 expression in fully kindled and sham control animals

| **Area** | **Ipsi-/Contralateral to electrode** | **Parameter** | **Difference between means ± SEM or medians (Hodges-Lehmann)** | **95 % CI** | **p-value** |
| --- | --- | --- | --- | --- | --- |
| **CA1** | Ipsilateral | Optical density | -0.01568 ± 0.008949 | -0.03429 to 0.002929 | 0.0943 |
|  |  | Labeled area | 0.04625 ± 1.183 | -2.407 to 2.500 | 0.9692 |
| **CA3** |  | Optical density | -0.008000 | -0.02400 to 0.006000 | 0.2468 |
|  |  | Labeled area | -0.8664 ± 1.457 | -3.888 to 2.156 | 0.5582 |
| **Hilus** |  | Optical density | 0.007000 | -0.004000 to 0.01900 | 0.2468 |
|  |  | Labeled area | -1.245 ± 1.055 | -3.438 to 0.9489 | 0.2512 |
| **Amygdala** | Contralateral | Optical density | 0.0009167 ± 0.005128 | -0.009717 to 0.01155 | 0.8598 |
|  |  | Labeled area | -2.268 ± 1.784 | -5.968 to 1.432 | 0.2169 |
| **Entorhinal cortex** | Ipsilateral | Optical density | 0.007333 ± 0.005493 | -0.004059 to 0.01873 | 0.1955 |
|  |  | Labeled area | -1.144 ± 1.491 | -4.236 to 1.947 | 0.4508 |
| **Piriform cortex** |  | Optical density | -0.001750 ± 0.006488 | -0.01520 to 0.01170 | 0.7899 |
|  |  | Labeled area | -1.009 ± 1.696 | -4.527 to 2.509 | 0.5579 |
| **Parietal cortex** |  | Optical density | -0.007833 ± 0.008226 | -0.02489 to 0.009226 | 0.3513 |
|  |  | Labeled area | -4.293 ± 3.790 | -12.15 to 3.568 | 0.2695 |
| **Perirhinal cortex** |  | Optical density | -0.004750 ± 0.005027 | -0.01518 to 0.005675 | 0.3550 |
|  |  | Labeled area | -2.777 ± 1.791 | -6.491 to 0.9369 | 0.1352 |
| **Lateral posterior nucleus** |  | Optical density | -0.002167 ± 0.006043 | -0.01470 to 0.01037 | 0.7234 |
|  |  | Labeled area | 0.3697 ± 2.084 | -3.952 to 4.692 | 0.8608 |
| **Mediodorsal nucleus** |  | Optical density | -0.002500 ± 0.005093 | -0.01306 to 0.008062 | 0.6284 |
|  |  | Labeled area | -1.002 ± 2.023 | -5.197 to 3.193 | 0.6253 |
| **Reticular nucleus** |  | Optical density | -0.006083 ± 0.004152 | -0.01469 to 0.002528 | 0.1571 |
|  |  | Labeled area | -1.097 ± 1.901 | -5.051 to 2.856 | 0.5700 |

Data are presented as mean ± SEM with 95% CI for normally distributed parameters or as median difference (Hodges-Lehmann estimation) with 95% CI for non-normally distributed parameters; normality was assessed using the Shapiro-Wilk test.

**Table SI6** Mean difference with 95% CI and p-value for the analysis of optical density and labeled area of Sigma-1 expression in kainate-treated and control animals at 2 d and 35 d after SE induction

| **Area** | **Ipsi-/Contralateral to electrode** | **Parameter** | **Mean difference** | **95 % CI** | **p-value** |
| --- | --- | --- | --- | --- | --- |
| **CA1 (2 d)** | Contralateral | Optical density | -0.008889 | -0.03188 to 0.01410 | 0.6100 |
|  |  | Labeled area | 0.8453 | -0.9135 to 2.604 | 0.4695 |
| **CA1 (35 d)** |  | Optical density | -0.005083 | -0.02807 to 0.01790 | 0.8488 |
|  |  | Labeled area | 0.9156 | -0.8827 to 2.714 | 0.4293 |
| **CA3 (2 d)** |  | Optical density | -0.02792 | -0.04517 to -0.01066 | 0.0010 |
|  |  | Labeled area | 3.878 | 1.415 to 6.341 | 0.0014 |
| **CA3 (35 d)** |  | Optical density | -0.003083 | -0.02034 to 0.01417 | 0.8983 |
|  |  | Labeled area | -0.4881 | -2.951 to 1.975 | 0.8765 |
| **Hilus (2 d)** |  | Optical density | -0.007583 | -0.02393 to 0.008766 | 0.4937 |
|  |  | Labeled area | 0.7759 | -0.6363 to 2.188 | 0.3756 |
| **Hilus (35 d)** |  | Optical density | 0.008379 | -0.008338 to 0.02510 | 0.4403 |
|  |  | Labeled area | 1.639 | 0.1953 to 3.083 | 0.0234 |
| **Amygdala (2 d)** | Ipsilateral | Optical density | 0.002667 | -0.01675 to 0.02208 | 0.9385 |
|  |  | Labeled area | 1.640 | -2.717 to 5.997 | 0.6252 |
| **Amygdala (35 d)** |  | Optical density | 0.001167 | -0.01825 to 0.02058 | 0.9879 |
|  |  | Labeled area | -1.081 | -5.342 to 3.180 | 0.8061 |
| **Entorhinal cortex (2 d)** |  | Optical density | -0.02017 | -0.03919 to -0.001144 | 0.0359 |
|  |  | Labeled area | 3.510 | -0.4113 to 7.432 | 0.0862 |
| **Entorhinal cortex (35 d)** |  | Optical density | -0.005333 | -0.02436 to 0.01369 | 0.7692 |
|  |  | Labeled area | 2.410 | -1.599 to 6.420 | 0.3124 |
| **Perirhinal cortex (2 d)** |  | Optical density | -0.01475 | -0.03616 to 0.006664 | 0.2219 |
|  |  | Labeled area | -0.4230 | -5.382 to 4.536 | 0.9757 |
| **Perirhinal cortex (35 d)** |  | Optical density | -0.004750 | -0.02616 to 0.01666 | 0.8480 |
|  |  | Labeled area | -0.4193 | -5.269 to 4.431 | 0.9751 |
| **Piriform cortex (2 d)** |  | Optical density | -0.01649 | -0.03873 to 0.005744 | 0.1771 |
|  |  | Labeled area | 1.393 | -2.256 to 5.042 | 0.6175 |
| **Piriform cortex (35 d)** |  | Optical density | -0.005917 | -0.02767 to 0.01583 | 0.7808 |
|  |  | Labeled area | -0.6025 | -4.252 to 3.047 | 0.9124 |
| **Parietal cortex (2 d)** | Contralateral | Optical density | 0.01050 | -0.01214 to 0.03314 | 0.4943 |
|  |  | Labeled area | 1.207 | -6.648 to 9.061 | 0.9237 |
| **Parietal cortex (35 d)** |  | Optical density | 0.01150 | -0.01114 to 0.03414 | 0.4314 |
|  |  | Labeled area | 1.929 | -5.926 to 9.783 | 0.8173 |
| **Reticular nucleus (2 d)** | Ipsilateral | Optical density | -0.004583 | -0.01989 to 0.01072 | 0.7417 |
|  |  | Labeled area | 1.112 | -3.117 to 5.341 | 0.7936 |
| **Reticular nucleus (35 d)** |  | Optical density | -0.01429 | -0.02960 to 0.001014 | 0.0709 |
|  |  | Labeled area | 0.8152 | -3.413 to 5.044 | 0.8827 |
| **Lateral posterior nucleus (2 d)** |  | Optical density | 0.005333 | -0.01143 to 0.02209 | 0.7139 |
|  |  | Labeled area | 3.511 | -0.6265 to 7.649 | 0.1084 |
| **Lateral posterior nucleus (35 d)** |  | Optical density | 0.01379 | -0.002968 to 0.03055 | 0.1226 |
|  |  | Labeled area | -0.8396 | -4.977 to 3.298 | 0.8710 |
| **Mediodorsal nucleus (2 d)** |  | Optical density | -0.003727 | -0.02558 to 0.01813 | 0.9068 |
|  |  | Labeled area | 1.378 | -3.040 to 5.796 | 0.7233 |
| **Mediodorsal nucleus (35 d)** |  | Optical density | 0.0006250 | -0.02075 to 0.02200 | 0.9971 |
|  |  | Labeled area | 1.478 | -2.939 to 5.896 | 0.6893 |

**Table SI7** Mean difference with 95% CI and p-value for the analysis of cell density of Sigma-1 expression in kainate-treated and control animals at 2 d and 35 d after SE-induction

| **Area** | **Ipsi-/Contralateral to electrode** | **Mean difference** | **95 % CI** | **p-value** |
| --- | --- | --- | --- | --- |
| **CA1 (2 d)** | Contralateral | -20,753 | -59,319 to 17,814 | 0.3907 |
| **CA1 (35 d)** |  | -20,843 | -59,409 to 17,724 | 0.3876 |
| **CA3 (2 d )** |  | -5,058 | -28,713 to 18,597 | 0.8577 |
| **CA3 (35 d)** |  | -14,571 | -37,706 to 8,564 | 0.2804 |
| **Hilus (2 d)** |  | -1,469 | -7,117 to 4,179 | 0.7976 |
| **Hilus (35 d)** |  | 2,052 | -3,595 to 7,700 | 0.6456 |
| **Entorhinal cortex (2 d)** | Ipsilateral | -3,763 | -10,101 to 2,575 | 0.3214 |
| **Entorhinal cortex (35 d)** |  | 676.3 | -5,661 to 7,014 | 0.9624 |

**Table SI8.** Correlation analysis between cell densisty and seizure parameters, as well as between ImageJ-based quantification (optical density and labeled area) and seizure parameters

| **Timepoint** | **Area** | **Comparison** | **Spearman r** | **p-value** | **n** | **95% CI** |
| --- | --- | --- | --- | --- | --- | --- |
| **2 d** | CA1 | SE duration vs. cell density | 0.07692 | 0.8171 | 12 | -0.5339 to 0.6350 |
|  |  | SE duration vs. OD | 0.5944 | 0.0457 | 12 | 0.01182 to 0.8757 |
|  |  | SE duration vs. LA | -0.7203 | 0.0106 | 12 | -0.9187 to -0.2313 |
|  | CA3 | SE duration vs. cell density | -0.2797 | 0.3789 | 12 | -0.7443 to 0.3673 |
|  |  | SE duration vs. OD | 0.5105 | 0.0936 | 12 | -0.1088 to 0.8443 |
|  |  | SE duration vs. LA | -0.5804 | 0.0521 | 12 | -0.8706 to 0.009540 |
|  | Hilus | SE duration vs. cell density | 0.3497 | 0.2662 | 12 | -0.2982 to 0.7770 |
|  |  | SE duration vs. OD | 0.5149 | 0.0894 | 12 | -0.1029 to 0.8460 |
|  |  | SE duration vs. LA | 0.2238 | 0.4851 | 12 | -0.4178 to 0.7164 |
|  | Entorhinal cortex | SE duration vs. cell density | 0.2937 | 0.3545 | 12 | -0.3540 to 0.7510 |
|  |  | SE duration vs. OD | 0.3497 | 0.2662 | 12 | -0.2982 to 0.7770 |
|  |  | SE duration vs. LA | -0.2657 | 0.4042 | 12 | -0.7375 to 0.3803 |
| **35 d** | CA1 | SEE frequency vs. cell density | 0.3222 | 0.3045 | 12 | -0.3261 to 0.7644 |
|  |  | SEE frequency vs. OD | 0.06655 | 0.8389 | 12 | -0.5413 to 0.6287 |
|  |  | SEE frequency vs. LA | 0.2460 | 0.4625 | 11 | -0.4319 to 0.7463 |
|  | CA3 | SEE frequency vs. cell density | 0.1401 | 0.6623 | 12 | -0.4866 to 0.6716 |
|  |  | SEE frequency vs. OD | -0.4095 | 0.1850 | 12 | -0.8032 to 0.2333 |
|  |  | SEE frequency vs. LA | -0.2557 | 0.4198 | 12 | -0.7325 to 0.3894 |
|  | Hilus | SEE frequency vs. Cell density | 0.1541 | 0.6302 | 12 | -0.4756 to 0.6794 |
|  |  | SEE frequency vs. OD | -0.1370 | 0.6856 | 11 | -0.6917 to 0.5194 |
|  |  | SEE frequency vs. LA | 0.5923 | 0.0588 | 11 | -0.03229 to 0.8842 |
|  | Entorhinal cortex | SEE frequency vs. cell density | 0.08757 | 0.7875 | 12 | -0.5262 to 0.6413 |
|  |  | SEE frequency vs. OD | 0.08056 | 0.8045 | 12 | -0.5313 to 0.6372 |
|  |  | SEE frequency vs. LA | 0.2642 | 0.4291 | 11 | -0.4159 to 0.7548 |

**OD** optical density; **LA** labeled area (%); **SE** status epilepticus; **SEE** spontaneous epileptiform events

1.	Racine, R.J., (1972) *Modification of seizure activity by electrical stimulation: II. Motor seizure.* Electroencephalography and Clinical Neurophysiology. **32**(3): p. 281-294, <https://doi.org/10.1016/0013-4694(72)90177-0>.

2.	GUNDERSEN, H.J.G., E.B.V. JENSEN, K. KIÊU, and J. NIELSEN, (1999) *The efficiency of systematic sampling in stereology — reconsidered.* Journal of Microscopy. **193**(3): p. 199-211, <https://doi.org/10.1046/j.1365-2818.1999.00457.x>.

3.	Bustin, S.A., J.M. Ruijter, M.J.B. van den Hoff, M. Kubista, M.W. Pfaffl, G.L. Shipley, N. Tran, S. Rödiger, A. Untergasser, R. Mueller, T. Nolan, M. Milavec, M.J. Burns, J.F. Huggett, J. Vandesompele, and C.T. Wittwer, (2025) *MIQE 2.0: Revision of the Minimum Information for Publication of Quantitative Real-Time PCR Experiments Guidelines.* Clinical Chemistry. **71**(6): p. 634-651, 10.1093/clinchem/hvaf043.
