# Supplementary material for: Sigma-1 Expression in Chronic Mouse Models of Temporal Lobe Epilepsy
Source: Mol Neurobiol. 2026 Aug 25;63(1):855. doi: 10.1007/s12035-026-06155-6 (PMC13506606; doi:10.1007/s12035-026-06155-6)
Supplement: Supplementary file 2 [file 12035_2026_6155_MOESM2_ESM.docx]

**Supplementary Blots**

**Related to Fig. 4** Specificity of the tested Sigma-1 antibodies in Western blotting:

Wild-type vs. Sigma1-knockout

**Panel A**

| Sigma-1 antibody #61994S | ß-tubulin |
| --- | --- |
|  |  |

**Panel B**

| Sigma-1 antibody ab53852 | ß-tubulin |
| --- | --- |
|  |  |

**Panel C**

| Sigma-1 antibody ab253192 | ß-tubulin |
| --- | --- |
|  |  |

**Panel D**

| Sigma-1 antibody BS-5111R | ß-tubulin |
| --- | --- |
|  |  |

**Panel E**

| Sigma-1 antibody HPA024701 | ß-tubulin |
| --- | --- |
|  |  |

**Panel F**

| Sigma-1 antibody MABN390 | ß-tubulin |
| --- | --- |
|  |  |

**Panel G**

| Sigma-1 antibody sc-137075 | ß-tubulin |
| --- | --- |
|  |  |

**Panel H**

| Sigma-1 antibody GTX115389 | ß-tubulin |
| --- | --- |
|  |  |

**Panel I**

| Sigma-1 antibody #727 | ß-tubulin |
| --- | --- |
|  |  |

**Panel J**

| Sigma-1 antibody HPA018002 | ß-tubulin |
| --- | --- |
|  |  |
